# Water-soluble host–guest complexes between fullerenes and a sugar-functionalized tribenzotriquinacene assembling to microspheres

**DOI:** 10.3762/bjoc.16.207

**Published:** 2020-10-14

**Authors:** Si-Yuan Liu, Xin-Rui Wang, Man-Ping Li, Wen-Rong Xu, Dietmar Kuck

**Affiliations:** 1 Key Laboratory of Advanced Materials of Tropical Island Resources of Ministry of Education, Hainan Provincial Key Laboratory of Fine Chemistry, Department of Chemistry, School of Science, Hainan University, Haikou, 570228, China; 2 Department of Chemistry, Center for Molecular Materials (CM2), Bielefeld University, 33615 Bielefeld, Germany

**Keywords:** fullerenes, host–guest systems, microspheres, supramolecular chemistry, tribenzotriquinacene

## Abstract

A sugar-functionalized water-soluble tribenzotriquinacene derivative bearing six glucose residues, **TBTQ-(OG)_6_**, was synthesized and its interaction with C_60_ and C_70_-fullerene in co-organic solvents and aqueous solution was investigated by fluorescence spectroscopy and ultraviolet-visible spectroscopy. The association stoichiometry of the complexes **TBTQ-(OG)_6_** with C_60_ and **TBTQ-(OG)_6_** with C_70_ was found to be 1:1 with binding constants of *K*_a_ = (1.50 ± 0.10) × 10^5^ M^−1^ and *K*_a_ = (2.20 ± 0.16) × 10^5^ M^−1^, respectively. The binding affinity between **TBTQ-(OG)_6_** and C_60_ was further verified by Raman spectroscopy. The geometry of the complex of **TBTQ-(OG)_6_** with C_60_ deduced from DFT calculations indicates that the driving force of the complexation is mainly due to the hydrophobic effect and to host–guest π–π interactions. Hydrophobic surface simulations showed that **TBTQ-(OG)_6_** and C_60_ forms an amphiphilic supramolecular host–guest complex, which further assembles to microspheres with diameters of 0.3–3.5 μm, as determined by scanning electron microscopy.

## Introduction

**Figure 1:**
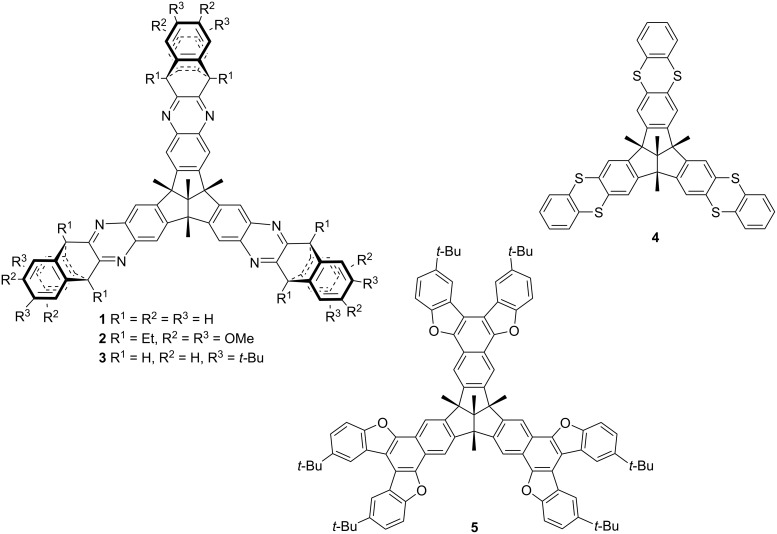
Selected TBTQ derivatives **1**–**5** that bind fullerenes in host–guest complexes.

In the field of supramolecular chemistry, host–guest association through noncovalent interactions is an interesting and exciting topic, especially for the encapsulation of various fullerenes, such as C_60_ and C_70_ [1–5]. It is generally accepted that good complexation of fullerenes requires host molecules with bowl or basket-like shapes, such as calixarenes [6], corannulenes [7–10], cyclodextrins [11–13], cyclotriveratrylenes [14–16], and similar macrocycles [17–20]. Tribenzotriquinacene (TBTQ) and its derivatives, owing to their unique rigid, *C*_3_*_v_*-symmetric, concave-convex trifuso-triindane skeleton that consists of three perfectly orthogonally oriented indane wings, bear a similar potential as molecular hosts. TBTQ hydrocarbons are chemically stable and offer various possibilities for functionalization. Therefore, TBTQ derivatives have attracted much attention since the first synthesis reported in 1984 [21–24]. The arene periphery of the TBTQ framework bears a great and variable potential for the efficient expansion of the small and relatively shallow cavity of the parent TBTQ hydrocarbons, thus allowing for the inclusion of large guest molecules, such as the fullerenes. Several TBTQ derivatives with extended cavities have been developed by us and other groups. Volkmer et al. designed a series of novel TBTQ-based receptors, **1**–**3**, and studied their binding affinities to C_60_ [25–28]. Georghiou et al. [29] synthesized the tris(thianthreno)-annelated triquinacene **4**, and Cao et al. [30] constructed the tris(naphtho)triquinacene **5**, bearing six annelated benzofuran units, and they investigated the supramolecular interaction of these hosts with fullerenes (Figure 1). All these TBTQ-based hosts were found to bind fullerenes in organic solvents with different strengths, as indicated by UV–vis or ^1^H NMR titration experiments. Moreover, easily accessible *C*_3_*_v_*-symmetrical sixfold hydroxy-functionalized TBTQ derivatives gain increasing attention for potential applications in host–guest recognition, such as gas storage and cationic complexation [31–33].

In the past years, the recognition of the biological and pharmaceutical relevance of fullerenes, such as their photodynamic activity, phototoxicity, HIV-1 protease inhibitor ability, and oxidative stability, has promoted the exploration of water-soluble fullerenes for biological use [34–35]. The extremely hydrophobic nature of fullerenes requires a strongly hydrophilic supramolecular host to achieve water solubility. It is important to note that host–guest research on TBTQ derivatives with fullerenes has been limited to organic media so far because of the poor solubility of their complexes in aqueous media. Therefore, the design, synthesis, and exploration of water-soluble TBTQ derivatives offer attractive possibilities for broadening the applications of the TBTQ structural motif. In the work presented here, we have introduced sugar motifs that possess good water solubility and biocompatibility [36–38] at the six outer peripheral positions of the TBTQ framework to provide, for the first time, a water-soluble TBTQ-based host bearing an extended cavity. The complexation of this host with C_60_ and C_70_-fullerene was investigated in co-organic solvents and in aqueous solution. The hydrophobic surface simulation of this host indicated the formation of a supra-amphiphilic system, which, as shown by scanning electron microscopy, further self-assembles into microspheres in the solid state. It is shown that the inclusion of fullerenes into the water-soluble TBTQ-based host greatly compensates for their water-repulsive nature and results in the formation of self-assembled microspheres that may have some potential for biological and pharmaceutical applications.

## Results and Discussion

**Scheme 1:**
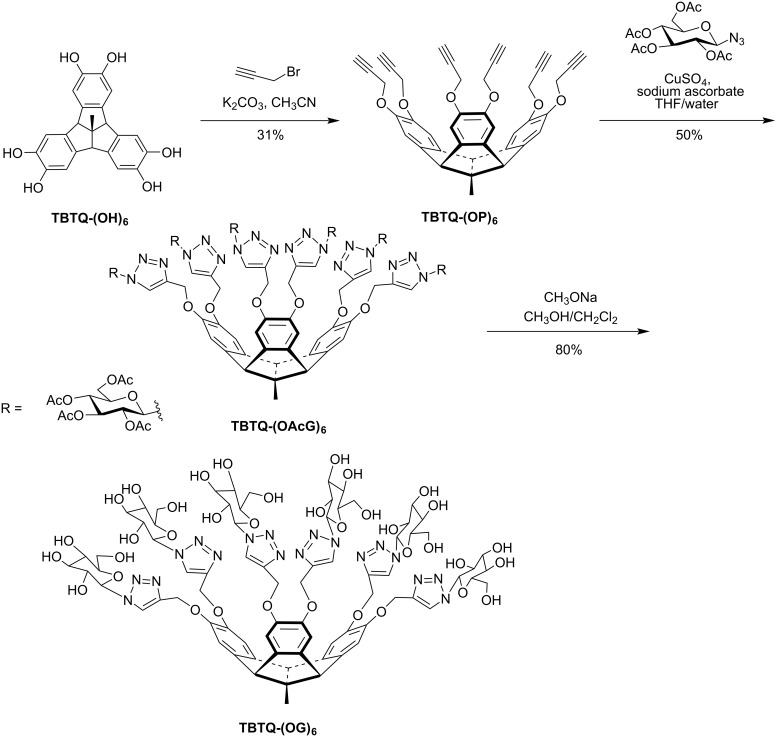
Synthetic route to **TBTQ-(OG)_6_**.

**Synthesis of the host TBTQ-(OG)_6_.** The sugar-functionalized host **TBTQ-(OG)_6_** was synthesized starting from the known compound **TBTQ-(OH)_6_** (Scheme 1) [31–33]. The reaction of **TBTQ-(OH)_6_** with propargyl bromide in the presence of potassium carbonate gave the hexakis-propargyl ether, **TBTQ-(OP)_6_**, in 31% yield. The subsequent CuAAC reaction with 1-azido-2,3,4,6-tetraacetylglucose, which was prepared according to the reported method [39], in the presence of Cu(I) as the catalyst afforded the acetyl-protected, sugar-functionalized derivative **TBTQ-(OAcG)_6_** in 50% yield. As expected, compound **TBTQ-(OAcG)_6_** exhibited good solubility in most organic solvents such as dichloromethane, chloroform, tetrahydrofuran, and DMSO, but it was insoluble in solvents like methanol, ethanol, and water. Compound **TBTQ-(OAcG)_6_** was finally deacetylated with sodium methoxide in methanol to afford the desired six-fold sugar-functionalized derivative **TBTQ-(OG)_6_** in 80% yield. The solubility of this compound was completely different from that of its acetylated precursor: it exhibited good solubility in DMF, DMSO, toluene/DMSO 1:1 (v/v) as well as in water.

**Structural characterization.** All synthesized compounds were fully characterized by ^1^H and ^13^C NMR spectroscopy and mass spectrometry (see Supporting Information File 1) and the data were found to be consistent with the proposed structures. For the hexakis-propargyl ether **TBTQ-(OP)_6_**, the signals at δ = 2.53 ppm in the ^1^H NMR spectrum and at δ = 78.98 and 76.07 ppm in the ^13^C NMR spectrum were attributed to the acetylene protons and carbons, respectively. The electrospray ionization (ESI) mass spectrum showed the most intense peak at *m*/*z* 641.1923, which corresponds to the [M + Na]^+^ molecular adduct ion and matches well with the calculated value (*m*/*z* 641.1935, Δ = −1.8 ppm). In addition, the corresponding [M + K]^+^ ion appears at *m*/*z* 657.1709 (Δ = + 5.3 ppm). The ^1^H NMR and ^13^C NMR spectra of compound **TBTQ-(OAcG)_6_** dissolved in DMSO-*d*_6_ are noteworthy because they reflect the diastereotopic environment of the different molecular building blocks. For example, the ^1^H NMR spectrum of **TBTQ-(OAcG)_6_** exhibits two singlet resonances at δ = 8.62 and δ = 8.58 ppm, each of which indicates three equivalent protons of the six triazole rings. Likewise, two singlets at δ = 7.33 and δ = 7.30 ppm are due to two sets of three equivalent arene protons of the TBTQ core [40]. The acetyl protons of the protected glucose residues appear as eight distinct resonances. The ^13^C NMR spectrum shows a similar splitting. The triazole carbons resonate at δ = 123.71 and δ = 123.69 ppm and at δ = 143.88 and δ = 143.79 ppm, indicating two sets of magnetically nonequivalent linkers. This is clearly a consequence of the prochiral nature of the TBTQ core. We also note that such splitting phenomenon was reported for neither six-fold sugar-functionalized cyclotriveratrylenes [41], which are directly comparable to **TBTQ-(OAcG)_6_**, nor for six-fold sugar-functionalized triptycene [42], and ten-fold sugar-functionalized pillar[5]arene [43]. Temperature-dependent ^1^H NMR spectroscopy of **TBTQ-(OAcG)_6_** in DMSO-*d*_6_ was carried out in the range of 20–70 °C and revealed a slight decrease of the splitting of the aromatic proton resonances with increasing temperature, but no coalescence. While most of the resonances did not shift significantly, the triazole pair of singlets was shifted to higher field by Δδ = −0.14 ppm (see Figure S2 in Supporting Information File 1). All these observations reflect the molecular *C*_3_-symmetry of **TBTQ-(OAcG)_6_** in solution and the presence of two diastereotopic sets of three magnetically equivalent tentacles at the rigid, prochiral TBTQ skeleton.

The mass spectrometric characterization of **TBTQ-(OAcG)_6_** turned out to be quite difficult. The MALDI-(+) mass spectrum (see Figure S10 and Table S1 in Supporting Information File 1), recorded with α-cyano-4-hydroxycinnamic acid (CHCA) as a matrix, exhibits the base peak at *m*/*z* 2879 along with an adjacent intense peak at *m*/*z* 2896, which are assigned to the [M + Na]^+^ and [M + K]^+^ molecular adduct ions, respectively. Unfortunately, attempts to perform accurate mass measurements were unsuccessful. However, the MALDI mass spectrum also shows the characteristic losses of up to at least three tentacle residues from both the [M + Na]^+^ and [M + K]^+^ molecular ions [44–46]. In contrast to the MALDI mass spectrum, the high-resolution ESI-(+) mass spectrum of **TBTQ-(OAcG)_6_** (see Figure S11 and Table S2) exhibits a sole peak group with the maximum component at *m*/*z* 1505.9690 and Δ(*m*/*z*) = 0.5, indicating the presence of doubly charged ions. The most intense peak is assigned to the doubly charged [M + 1] isotopolog of the molecular adduct [M + 6H_2_O + 2Na]^2+^, the theoretical value of which is calculated to be *m*/*z* 1505.9611 (Δ = + 5.2 ppm). The surprising presence of six equivalents of water points to the formation of strong hydrogen bonds between the sugar ester bonds of **TBTQ-(OAcG)_6_** and the water molecules as guests. However, the origin of this intriguing observation associated with the attachment of just two sodium cations requires further studies. Despite the unusual mass spectrometric behavior of the compound, the combined spectroscopic evidence strongly supports the identity of **TBTQ-(OAcG)_6_**.

After deprotection of the glucose units, the acetyl signals disappeared in the ^1^H and ^13^C NMR spectra of the target compound **TBTQ-(OG)_6_**. The ^1^H NMR spectrum showed characteristic resonances for the TBTQ core, the six triazole rings and the six glucose units but also some broadened signals. In contrast to the acetylated precursor, splitting of the proton signals is almost completely absent. At first glance, the ^13^C NMR spectrum exhibited only 14 of the 15 resonances expected for a *C*_3_-symmetric structure. However, a closer inspection revealed again a tiny splitting of several resonances, e.g., of those of the triazole rings at δ = 124.21 and δ = 124.24 ppm and at δ = 142.80 and δ = 142.82 ppm, but also for those of the benzene rings of the TBTQ core. Even the six resonances of the glucose carbons appear to split into two signals (e.g., at δ = 79.96 and δ = 79.98 ppm. Temperature-dependent ^1^H NMR spectroscopy of **TBTQ-(OG)_6_** again revealed a similar upfield shift of the triazole resonance as that observed for the precursor (Δδ = −0.13 ppm). Even more significant upfield shifts were found for the resonances of the glucose protons with exception of the doublet of the glycosidic protons.

The MALDI-(+) mass spectrum of **TBTQ-(OG)_6_** (see Figure S14 and Table S3, Supporting Information File 1) exhibits dominating [M + Na]^+^ and [M + K]^+^ molecular ion peaks at *m*/*z* 1871 and *m*/*z* 1887, respectively, in analogy to the spectrum of the precursor compound. Also, characteristic fragment ions peaks of minor intensity appear *m*/*z* 1709 and *m*/*z* 1628, indicating the elimination of a glucose unit as C_6_H_10_O_5_ (162 u) and, respectively, the loss of the entire tentacle as an C_9_H_14_N_3_O_5_^·^ radical (244 u) from the [M + Na]^+^ ion followed by H atom transfer. Again, accurate mass measurements were not obtained. As another surprise, the high-resolution ESI-(−) mass spectrum of **TBTQ-(OG)_6_** (see Figure S15 and Table S4, Supporting Information File 1) exhibits a prominent [M − 2 H]^2−^ peak at *m*/*z* 923.3014 which is consistent with the calculated value (*m*/*z* 923.3045, Δ = −3.3 ppm). Together with the ^1^H and ^13^C NMR spectra this unambiguously confirms the successful synthesis of the host compound **TBTQ-(OG)_6_**.

**Figure 2:**
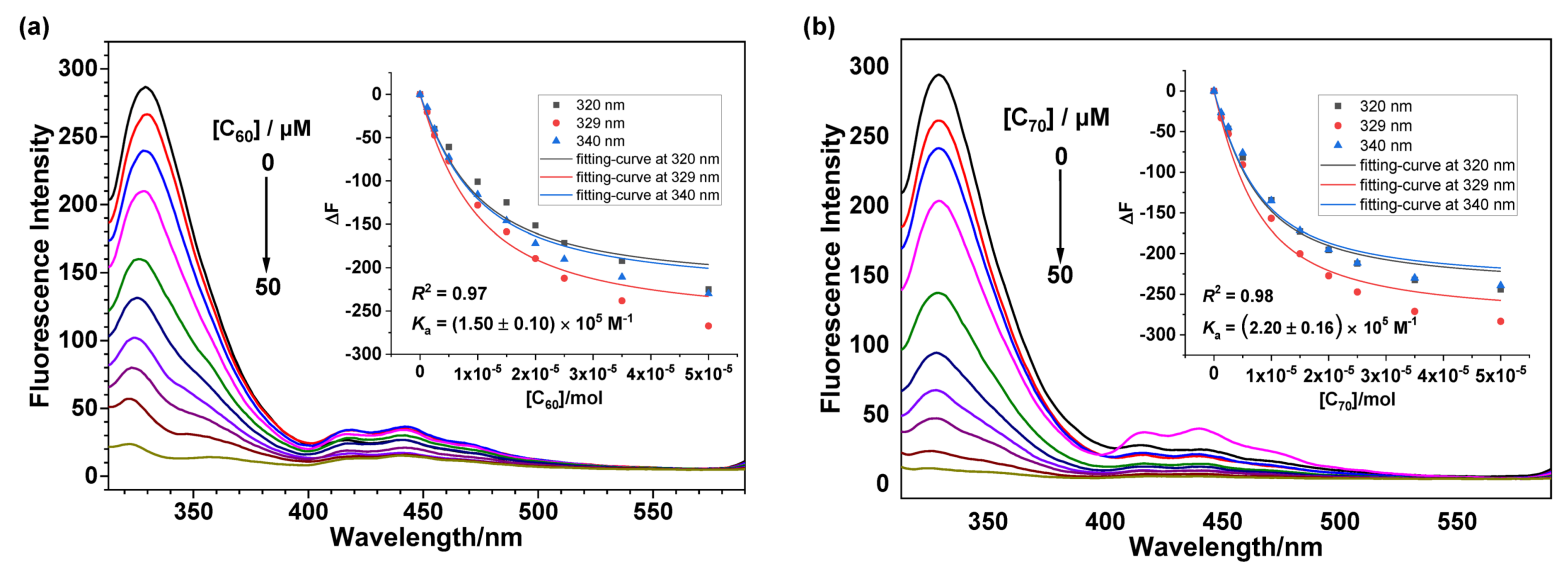
Fluorescence spectra of **TBTQ-(OG)_6_** (5.0 × 10^−6^ M) with varying concentrations of (a) C_60_ and (b) C_70_ (0.0, 1.25, 2.5, 5.0, 10.0, 15.0, 20.0, 25.0, 35.0, 50.0 ×10^−6^ M) in toluene/DMSO 1:1 (v/v) (λ_ex_ = 294 nm). The insets show the respective plots of Δ*F* vs [C_60_] and [C_70_] at 320 nm, 329 nm and 340 nm (the solid line was obtained from the global nonlinear curve fitting).

**Complexation of TBTQ-(OG)_6_ with fullerenes.** In order to investigate the host–guest relationship between **TBTQ-(OG)_6_** and fullerenes, fluorescence titration experiments were performed in toluene/DMSO 1:1 (v/v), in which both the host and the guest components could be dissolved, instead of in water. As shown in Figure 2, the spectrum of **TBTQ-(OG)_6_** exhibits an emission maximum peak at 329 nm upon excitation at 294 nm. This emission band is probably due to the formation of the ground-state dimer by interaction between the benzene rings upon excitation or excimer emission caused by the interaction between the aromatic rings [41,43]. As the concentration of the fullerenes C_60_ and C_70_ increase, the emission is significantly quenched, indicating the photoinduced energy transfer from **TBTQ-(OG)_6_** to the fullerenes [47–48]. Molar ratio plots (see Figure S16, Supporting Information File 1) on the basis of the fluorescence titration experiments suggested a 1:1 stoichiometric ratio of both **TBTQ-(OG)_6_** 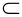 C_60_ and **TBTQ-(OG)_6_** 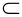 C_70_, and the association constants were calculated to be *K*_a_ = (1.50 ± 0.10) × 10^5^ M^−1^ and *K*_a_ = (2.20 ± 0.16) × 10^5^ M^−1^, respectively, using a global nonlinear curve fitting method (Figure 2) [49]. The slightly stronger association affinity of C_70_-fullerene may be attributed to its larger size and surface area as compared to C_60_-fullerene [50].

**Figure 3:**
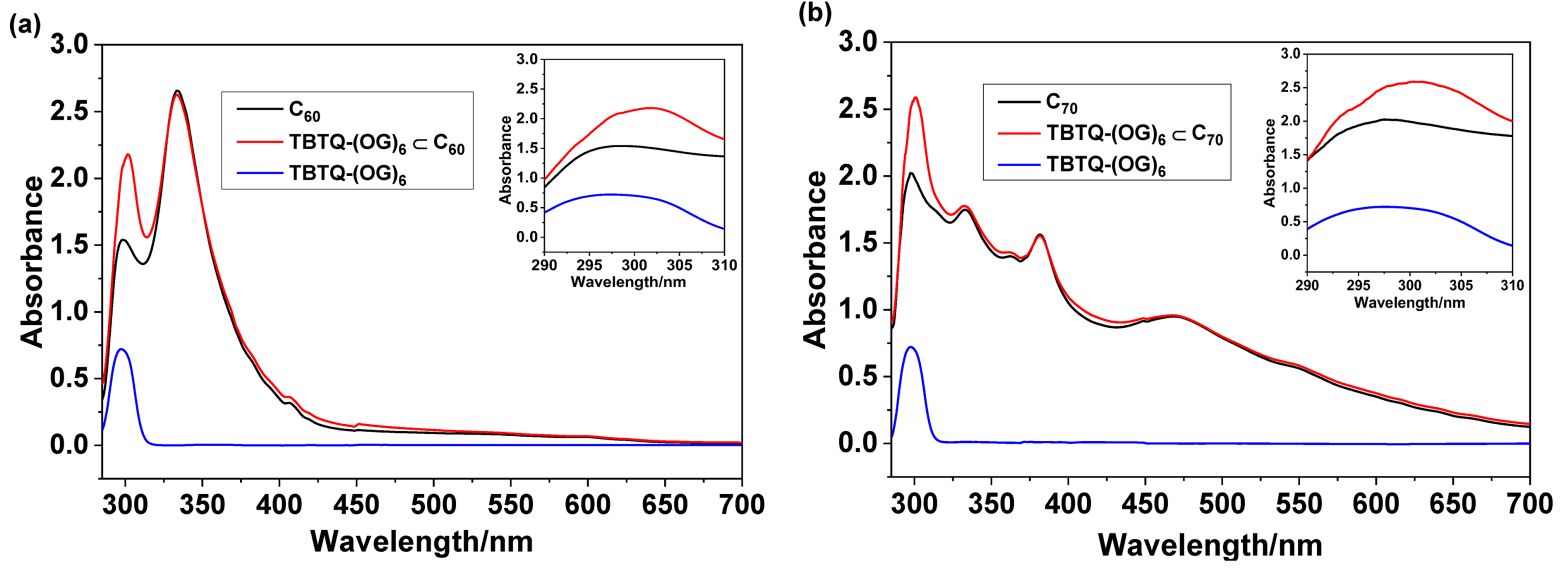
Absorption spectra of (a) **TBTQ-(OG)_6_** 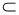 C_60_ [**TBTQ-(OG)_6_**: 50 μM; C_60_: 50 μM] and (b) **TBTQ-(OG)_6_** 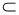 C_70_ [**TBTQ-(OG)_6_**: 50 μM; C_70_: 50 μM] in toluene/DMSO 1:1 (v/v) after centrifugation. The insets show the magnified partial UV curves in the range of 290–310 nm.

**Figure 4:**
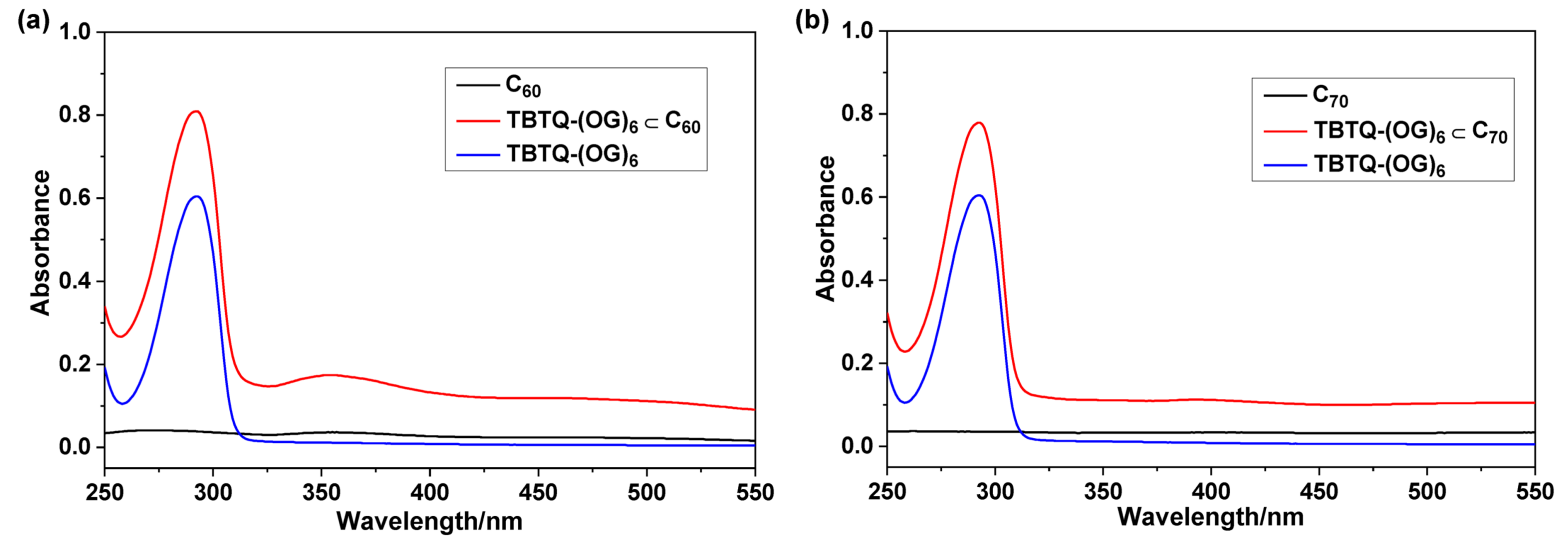
Absorption spectra of (a) **TBTQ-(OG)_6_** 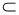 C_60_ [**TBTQ-(OG)_6_**: 50 μM; C_60_: 50 μM] and (b) **TBTQ-(OG)_6_** 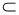 C_70_ [**TBTQ-(OG)_6_**: 50 μM; C_70_: 50 μM] in water after centrifugation.

The complexation between **TBTQ-(OG)_6_** and fullerenes was also examined by UV–vis spectroscopy in both toluene/DMSO 1:1 (v/v) and water. Figure 3a shows the UV–vis spectra of **TBTQ-(OG)_6_**, C_60_ and **TBTQ-(OG)_6_** 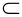 C_60_ in toluene/DMSO. **TBTQ-(OG)_6_** absorbs at 297 nm and C_60_ exhibits two absorption peaks at 298 nm and 333 nm. When C_60_ was mixed with **TBTQ-(OG)_6_** in a 1:1 molar ratio, the absorption of C_60_ at 298 nm was slightly shifted to 302 nm. Similarly, the absorption of C_70_ at 298 nm was shifted to 301 nm after mixing the fullerenes with **TBTQ-(OG)_6_** in the same molar ratio (Figure 3b). In aqueous solution, **TBTQ-(OG)_6_** showed an absorption at 292 nm and, as expected, practically no absorption was observed for C_60_ and C_70_ due to their poor solubility (Figure 4a and Figure 4b). However, 1:1 molar mixture of **TBTQ-(OG)_6_** and C_60_ in water exhibited an increased absorption at 292 nm and generated a new absorption band at 354 nm (Figure 4a). This observation is attributed to the increased solubility of C_60_ in water due to formation of the host–guest complex with **TBTQ-(OG)_6_**. A similar absorption behavior was observed for **TBTQ-(OG)_6_** 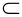 C_70_ (Figure 4b). The dispersibility of higher amounts of fullerenes in water in the presence of **TBTQ-(OG)_6_** was further assessed at different rest times after sonication for 10 min. As illustrated in Figure S17 (Supporting Information File 1), the pristine C_60_ and C_70_-fullerenes precipitated rapidly within 5 min. In contrast, the **TBTQ-(OG)_6_** 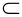 C_60_ and **TBTQ-(OG)_6_** 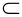 C_70_ complexes showed improved water dispersibility of the fullerenes, which was maintained even after 20 days. The maximum solubility of C_60_ in water was measured to be about 0.14 mg/mL in the presence of ten equivalents of **TBTQ-(OG)_6_**, while the maximum solubility of C_70_ was about 0.17 mg/mL.

**Figure 5:**
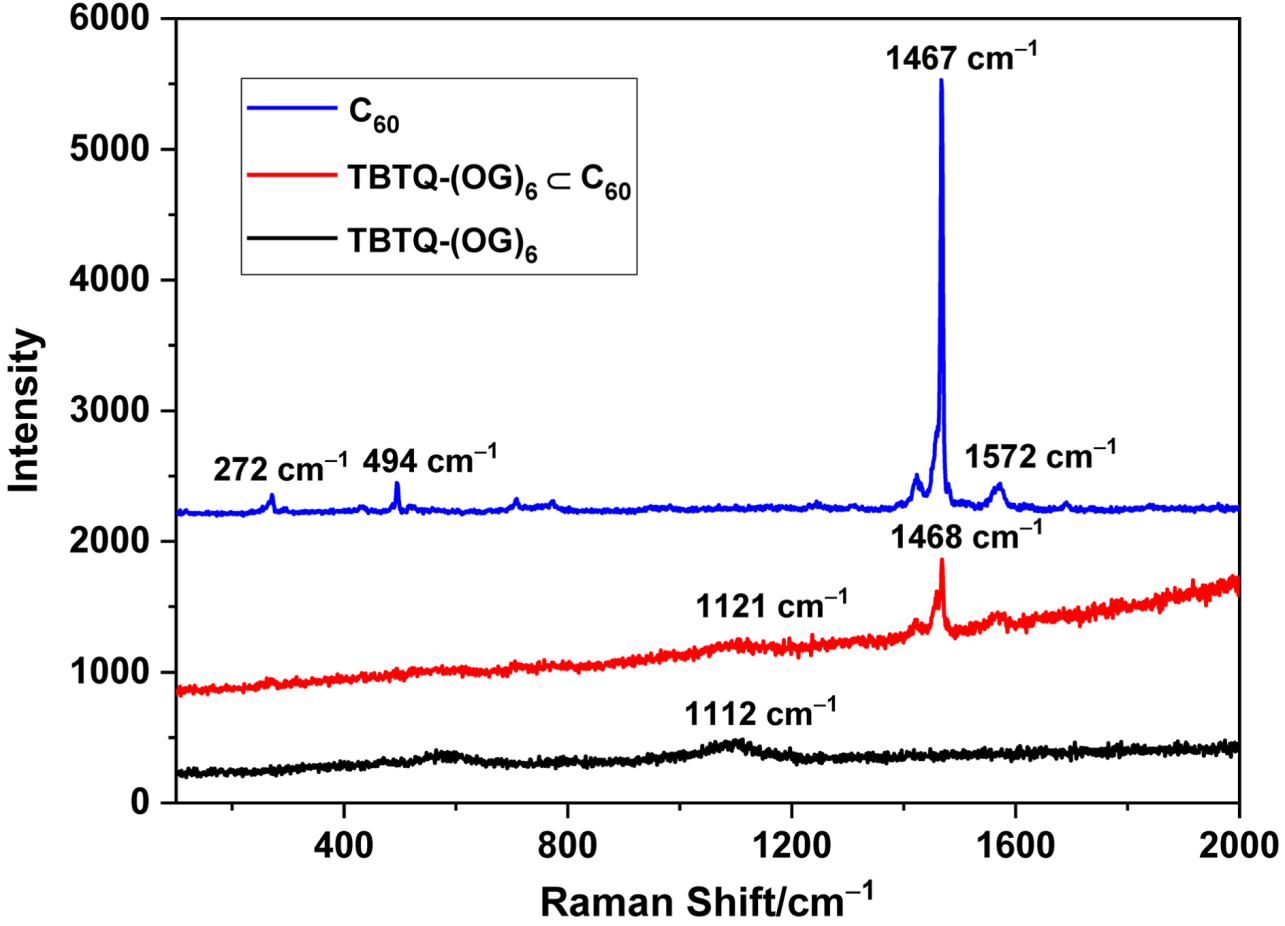
Raman spectra of **TBTQ-G_6_**, C_60_ and **TBTQ-G_6_** 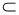 C_60_. Sample solutions of **TBTQ-(OG)_6_** (50 μM) and **TBTQ-(OG)_6_** (50 μM) 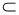 C_60_ (250 μM) were ultrasonicated for 10 min and then centrifuged to afford the supernatant, drops of which were dried on a slide glass. C_60_ was tested in powder form on a slide glass.

Raman spectroscopy has proven to be a useful tool for the characterization of carbon nanomaterials [51–52]. The Raman spectra of **TBTQ-(OG)_6_**, C_60_ and **TBTQ-(OG)_6_** 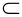 C_60_ are displayed in Figure 5. The **TBTQ-(OG)_6_** shows a weak peak at 1112 cm^−1^. The pristine C_60_-fullerene shows characteristic peaks at 494 cm^−1^ (A_g_-breathing mode), 1467 cm^−1^ (A_g_-pentagonal pinch mode), as well as signals at 272 cm^−1^ and 1572 cm^−1^ (H_g_ modes) [53]. The Raman spectrum of **TBTQ-(OG)_6_** 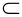 C_60_ clearly shows the presence of **TBTQ-(OG)_6_** and fullerene, and the slight shift of the peaks further indicates the successful complexation of **TBTQ-(OG)_6_** and C_60_.

**Figure 6:**
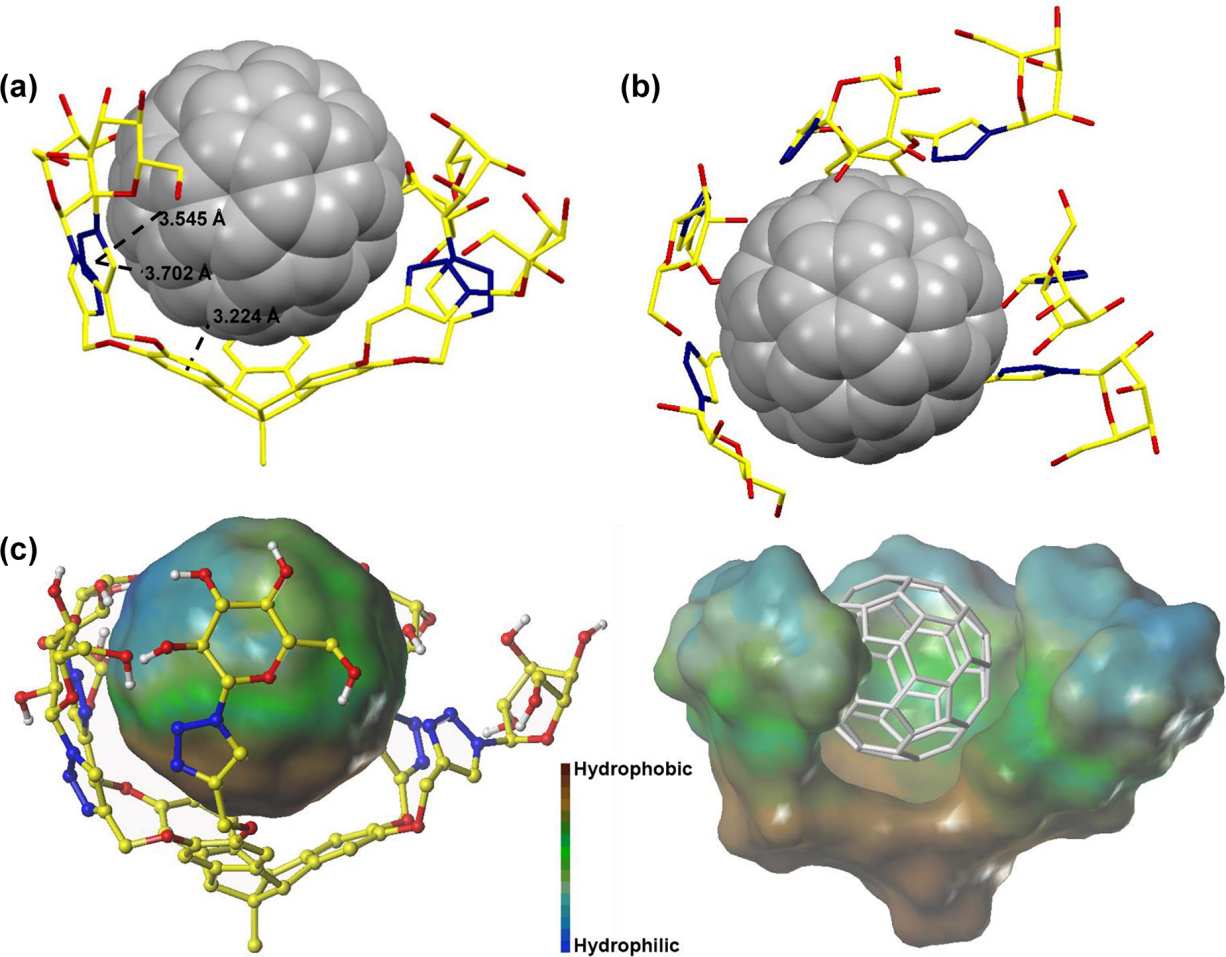
Molecular model of the complex **TBTQ-(OG)_6_** 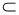 C_60_ in water, as generated by DFT calculations. (a) Side-view; (b) top-view; (c) hydrophobic surface diagram. In part, H atoms were omitted for clarity (yellow: C, red: O, blue: N, white: H for **TBTQ-(OG)_6_**; silver grey: C for C_60_).

**Simulations of complex TBTQ-(OG)_6_** 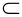 **C_60_ in water.** In spite of numerous attempts, we failed to obtain good-quality crystals to determine the binding conformation of complex **TBTQ-(OG)_6_** 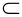 C_60_ by X-ray diffraction. Therefore, the optimized geometry of the 1:1 complex of **TBTQ-(OG)_6_** 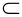 C_60_ in water was simulated by density functional theory (DFT) calculations at the B3LYP/6-31G(d) level of theory, which was completed with the aid of Molclus, MOPAC, and ORCA 4.1.0 programs [54–56]. As shown in Figure 6, C_60_-fullerene can be embedded between the six arms of **TBTQ-(OG)_6_** and the distance between the center of one benzene ring of the host and the closest five-membered ring of the guest was calculated to be 3.224 Å. The distances between the center of a triazole ring of **TBTQ-(OG)_6_** and the adjacent five and six-membered rings of C_60_ were calculated to be 3.541 and 3.702 Å, respectively. These results suggest significant host–guest π–π interactions between **TBTQ-(OG)_6_** and C_60_ [57]. In order to intuitively assess the hydrophilicity and hydrophobicity of the complex, the hydrophobic surface diagram was generated and is reproduced in Figure 6c. It is evident that **TBTQ-(OG)_6_** provides a strongly hydrophobic region at the inner bottom. Thus, the hydrophobic effect is assumed to be an important driving force for the formation of the complex **TBTQ-(OG)_6_** 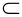 C_60_.

**Figure 7:**
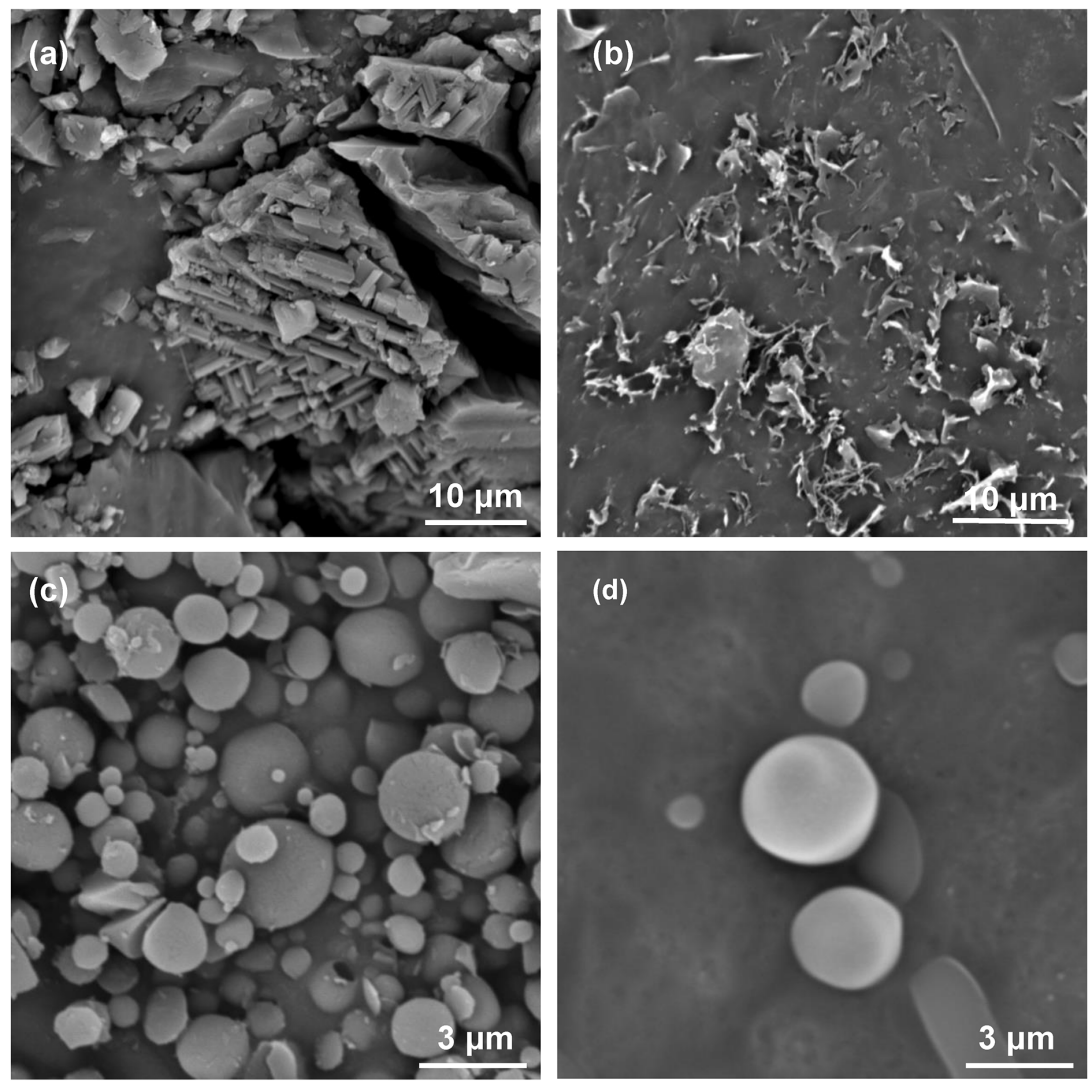
SEM images of (a) C_60_; (b) **TBTQ-(OG)_6_**; (c) and (d) **TBTQ-(OG)_6_** 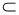 C_60_ (C_60_: 1.4 mM; **TBTQ-(OG)_6_:** 1.4 mM in water, samples were freeze-dried and gold-sputtered before imaging).

**Surface morphologies.** The surface morphologies of C_60_, **TBTQ-(OG)_6_**, and **TBTQ-(OG)_6_** 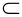 C_60_ were investigated by scanning electron microscopy (SEM). As shown in Figure 7a and 7b, respectively, the SEM image of C_60_-fullerene displays cylindrical nanotubes and that of **TBTQ-(OG)_6_** does not indicate any definite shape. However, well-dispersed microspheres with diameters of 0.3–3.5 μm were observed for the complex **TBTQ-(OG)_6_** 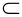 C_60_. Obviously, these aggregates form by further assembly of the supra-amphiphilic host–guest systems, as suggested in Figure 6c, due to the hydrophobic interactions governing the association behavior of **TBTQ-(OG)_6_** 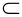 C_60_ in water.

## Conclusion

In summary, we have successfully synthesized a six-fold sugar-functionalized, water soluble tribenzotriquinacene derivative, **TBTQ-(OG)_6_**, which significantly improves the solubility of C_60_ and C_70_-fullerenes in water. The target compound and its peracetylated precursor **TBTQ-(OAcG)_6_** have been characterized in detail by NMR spectroscopy and mass spectrometry. The host–guest complexation of **TBTQ-(OG)_6_** with C_60_ and C_70_ takes place in a 1:1 molar ratio in both cases and with association constants of *K*_a_ = (1.50 ± 0.10) × 10^5^ M^−1^ and *K*_a_ = (2.20 ± 0.16) × 10^5^ M^−1^, respectively. It is suggested that the formation of the host–guest complexes is primarily due to hydrophobic effects and π–π interactions. The **TBTQ-(OG)_6_** 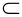 C_60_ complex was found to further assemble to microspheres with diameters of 0.3–3.5 μm. The inclusion complexation between **TBTQ-(OG)_6_** and fullerenes in aqueous solution may shed light on potential future applications of fullerenes in biological and pharmaceutical areas.

## Experimental

**General information.** All commercially available reagents were used as received unless otherwise stated. Anhydrous solvents were collected from a Mikrouna Solv Purer G3 solvent purification system. The ^1^H NMR and ^13^C NMR spectra were recorded on a 400 MHz Bruker NMR spectrometer and chemical shifts were reported in ppm (δ). The fluorescence spectra were measured on a F97Pro fluorescence spectrophotometer (LengGuang Tech, Shanghai, China) and UV–vis spectra were measured on a UV-3300PC spectrometer (Mapada Instruments Co., Ltd, Shanghai, China). Mass spectra were recorded using either electrospray ionization (ESI) on a LCMS-IT-TOF instrument (Shimadzu, Kyoto, Japan) or a matrix-assisted laser desorption/ionization (MALDI) on a RapifleX MALDI Tissuetyper system (Bruker Daltonik GmbH, Bremen, Germany). Raman spectra were recorded on an inVia Reflex confocal Raman microscope (Renishaw plc, Wotton-under-Edge, UK) by dropping the sample solutions of **TBTQ-(OG)_6_** and **TBTQ-(OG)_6_** 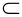 C_60_ onto a slide glass with subsequent air drying. C_60_ was tested in powder form on a slide glass. Ultrasonic mixing was performed with a 100 W ultrasonic cleaner. The surface morphology was investigated on a Phenom ProX scanning electron microscopy (SEM, Phenom World, Eindhoven, Netherlands). Samples were prepared from the freeze-dried aqueous solution/suspension of **TBTQ-(OG)_6_**, C_60_, and **TBTQ-(OG)_6_** 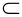 C_60_, and then gold-sputtered prior to imaging. Freeze drying was conducted on a Scientz-18N freeze dryer (Scientz Biotech, Zhejiang, China).

**Synthesis of TBTQ-(OP)_6_.** A mixture of **TBTQ-(OH)_6_** (2.17 g, 5.56 mmol), potassium carbonate (9.23 g, 66.8 mmol), and propargyl bromide (5.40 g, 44.5 mmol, 98 wt %) in acetonitrile (100 mL) was stirred and heated under a nitrogen atmosphere at 70 °C for 12 h. The mixture was then cooled to room temperature and the suspension was filtered, and washed with acetonitrile. The resulting filtrate was evaporated under vacuum to afford the crude product as yellow solid. Further recrystallization from dichloromethane/ethanol provided the pure product as pale-yellow solid (1.06 g, 1.71 mmol, 31%). Mp 197.3–197.4 °C; ^1^H NMR (400 MHz, CDCl_3_) δ 7.09 (s, 6H, H^Ar^), 4.72 (dd, *J* = 3.7 Hz, *J* = 2.4 Hz, 12H, OCH_2_), 4.32 (s, 3H, Ar_2_CH), 2.53 (t, *J* = 2.3 Hz, 6H, C≡CH), 1.68 (s, 3H, CH_3_); ^13^C NMR (100 MHz, CDCl_3_) 147.87, 138.88, 111.49, 78.98, 76.07, 63.51, 63.12, 57.54, 27.47; HRESIMS (*m*/*z*): [M + Na]^+^ calcd for C_41_H_30_NaO_6_^+^, 641.1935; found, 641.1923 (Δ = −1.8 ppm); [M + K]^+^ calcd for C_41_H_30_KO_6_^+^, 657.1674; found 657.1709 (Δ = +5.3 ppm).

**Synthesis of TBTQ-(OAcG)_6_.** A mixture of **TBTQ-(OP)_6_** (201 mg, 0.33 mmol), 1-azido-2,3,4,6-tetraacetylglucose (1.48 g, 3.96 mmol), copper(II) sulfate pentahydrate (52 mg, 0.21 mmol), and sodium ascorbate (28 mg, 0.14 mmol) in tetrahydrofuran/water cosolvent 2:1 (10 mL, v/v) was stirred vigorously under nitrogen in the dark at 60 °C for 24 h. Then, the solvent was removed under reduced pressure and water (25 mL) was added to the mixture, which was extracted with ethyl acetate (3 × 15 mL). The combined organic layers were dried over anhydrous magnesium sulfate and concentrated to dryness. The crude residue obtained was purified by silica gel column chromatography (petroleum ether/ethyl acetate 1:10; *R*_f_ = 0.6) to afford **TBTQ-(OAcG)_6_** as off-white solid (470 mg, 0.16 mmol, 50%). Mp 141–148 °C; ^1^H NMR (400 MHz, DMSO-*d*_6_, 20 °C) δ 8.62 (s, 3H), 8.58 (s, 3H), 7.33 (s, 3H), 7.30 (s, 3H), 6.37 (t, *J* = 5.6 Hz, 6H), 5.73–5.67 (m, 6H), 5.57–5.53 (m, 6H), 5.24–5.15 (m, 18H), 4.36 (m, 6H), 4.19 (s, 3H), 4.18–4.13 (m, 6H), 4.09–4.06 (m, 6H), 2.038 (s, 9H), 2.033 (s, 9H), 1.971 (s, 9H), 1.967 (s, 9H), 1.963 (s, 9H), 1.94 (s, 9H), 1.75 (s, 9H), 1.66 (s, 9H), 1.54 (s, 3H); ^13^C NMR (100 MHz, DMSO-*d*_6_) δ 170.04, 170.02, 169.59, 169.39, 169.36, 168.52, 168.51, 147.78, 147.76, 143.88, 143.79, 137.74, 123.71, 123.59, 110.56, 83.98, 83.91, 73.37, 73.35, 72.21, 72.19, 70.09, 67.54, 67.44, 62.57, 62.46, 62.20, 62.10, 61.72, 61.61, 59.77, 27.33, 20.43, 20.40. 20.38, 20.22, 19.80, 19.64; MALDI–MS (CHCA, *m*/*z*): 2879.5 (72), 2880.5 (100, both [M + Na]^+^), 2895.5 (24), 2896.5 (35), both [M + K]^+^), 2468.4 (39), 2469.4 (48), 2484.4 (16), 2485.4 (17), 2057.3 (29), 2058.3 (28), 2073.3 (13), 2074.3 (13), 1645.2 (8), 1646.2 (11), 1661.3 (5), 1662.3 (6)%; HRESIMS (*m*/*z*): [M + 6H_2_O + 2Na]^2+^ calcd for ^12^C_125_^1^H_156_^14^N_18_^23^Na_2_^16^O_66_^2+^, 1505.4594; found, 1505.4723 (Δ = +8.5 ppm); [M + 1] calcd for the ion containing one heavier isotope (mainly ^13^C_1_^12^C_124_^1^H_156_^14^N_18_^23^Na_2_^16^O_66_^2+^) [M + 6H_2_O + 2Na]^2+^, 1505.9611; found, 1505.9690 (Δ = +5.2 ppm).

**Synthesis of TBTQ-(OG)_6_.** To a solution of **TBTQ-(OAcG)_6_** (0.95 g, 0.33 mmol) in methanol (60 mL), sodium methoxide (about 1 mL) was added to adjust the pH value to 11. The mixture was stirred at room temperature for 12 h and then the suspension was filtered and washed with methanol. The filtrate was concentrated and purified by a Biogel P_2_ column to give **TBTQ-(OG)_6_** as colorless solid (0.49 g, 0.26 mmol, 80%). Mp 226–227 °C; ^1^H NMR (400 MHz, DMSO-*d*_6_, 20 °C) δ 8.46 (s, 6H), 7.47 (s, 6H), 5.56 (d, *J* = 6.1 Hz, 6H), 5.44–5.41 (dd, *J* = 4.4 Hz, *J* = 6.3 Hz, 6H), 5.29 (d, *J* = 3.2 Hz, 6H), 5.21–5.19 (m, 12 H), 5.17 (d, *J* = 3.7 Hz, 6 H), 4.69–4.66 (m, 6H), 4.25 (s, 3H), 3.81–3.76 (m, 6H), 3.68–3.64 (m, 6H), 3.44–3.42 (m, 12H), 3.40–3.36 (m, 6H), 3.25–3.22 (m, 6H), 1.61 (s, 3H); ^13^C NMR (100 MHz, DMSO-*d*_6_) δ 147.81, 147.77, 142.82, 142.80, 137.44, 124.24, 124.21, 109.88, 87.52, 79.98, 79.96, 76.95, 72.03, 69.50, 62.66, 62.60, 61.87, 61.78, 60.72, 27.53; MALDI–MS (CHCA, *m*/*z*): 1871.1 (95), 1872.1 (100, both [M + Na]^+^), 1887.1 (20), 1888.0 (20, both [M + K]^+^), 1709.1 (20), 1710.1 (21), 1628.1 (28), 1629.1 (24) %; HRESIMS (negative mode, *m*/*z*): [M – 2 H]^2−^ calcd for C_77_H_94_N_18_O_36_^2−^, 923.3045; found, 923.3014 (Δ = −3.3 ppm).

## Supporting Information

File 1 ^1^H NMR, ^13^C NMR spectroscopy, and mass spectrometry of all new compounds, and the *xyz* coordinates (in Å) of the complex of **TBTQ-(OG)_6_** with C_60_.
