# Supplementary material for: Water-soluble host–guest complexes between fullerenes and a sugar-functionalized tribenzotriquinacene assembling to microspheres
Source: Beilstein J Org Chem. 2020 Oct 14;16:2551–61. doi: 10.3762/bjoc.16.207 (PMC7590625; doi:10.3762/bjoc.16.207)
Supplement: File 1 — 1H NMR, 13C NMR spectroscopy, and mass spectrometry of all new compounds, and the xyz coordinates (in Å) of the complex of TBTQ-(OG)6 with C60. [file Beilstein_J_Org_Chem-16-2551-s001.pdf]

BEILSTEIN JOURNAL OF ORGANIC CHEMISTRY

Supporting Information

for

Water-soluble host–guest complexes between fullerenes and a sugar-functionalized tribenzotriquinacene assembling to microspheres

Si-Yuan Liu, Xin-Rui Wang, Man-Ping Li, Wen-Rong Xu and Dietmar Kuck

Beilstein J. Org. Chem. **2020,** *16,* 2551–2561. doi:10.3762/bjoc.16.207

^{1}H NMR, ^{13}C NMR spectroscopy, and mass spectrometry of all new compounds, and the *xyz* coordinates (in Å) of the complex of TBTQ-$(OG)_6$ with C_{60}

License and Terms: This is a supporting information file under the terms of the Creative Commons Attribution License (https://creativecommons.org/licenses/by/4.0). Please note that the reuse, redistribution and reproduction in particular requires that the authors and source are credited.
The license is subject to the *Beilstein Journal of Organic Chemistry* terms and conditions: (https://www.beilstein-journals.org/bjoc)

Table of contents

Figure S1: Enantiotopic triazole protons in parent (*M*)- and (*P*)-enantiomers……………S2

Figure S2: ^{1}H NMR spectra of **TBTQ-(OAcG)$_6$** at different temperatures...................... S2

Figure S3: ^{1}H NMR spectra of **TBTQ-(OG)$_6$** at different temperatures S3

Figure S4: Blank ^{1}H NMR spectra (water in DMSO-d_6) at different temperatures S4

Figure S5: ^{1}H NMR spectrum of **TBTQ-(OP)$_6$**... S5

Figure S6: ^{13}C NMR spectrum of **TBTQ-(OP)$_6$**.. S5

Figure S7: ESI-HRMS spectrum of **TBTQ-(OP)$_6$** S6

Figure S8: ^{1}H NMR spectrum of **TBTQ-(OAcG)$_6$**.. S7

Figure S9: ^{13}C NMR spectrum of **TBTQ-(OAcG)$_6$**... S7

Figure S10: MALDI mass spectrum of **TBTQ-(OAcG)$_6$**.. S8

Table S1: Extracted data from the MALDI mass spectrum of **TBTQ-(OAcG)$_6$**............... S9

Figure S11: ESI-HRMS spectrum of **TBTQ-(OAcG)$_6$**... S10

Table S2: Abundance pattern of the [M + 6 H_2O + 2 Na]$^{2+}$ ions from the
ESI mass spectrum of **TBTQ-(OAcG)$_6$** (low resolution).............................. S10

Figure S12: ^{1}H NMR spectrum of **TBTQ-(OG)$_6$**...S11

Figure S13: ^{13}C NMR spectrum of **TBTQ-(OG)$_6$** S11

Figure S14: MALDI mass spectrum of **TBTQ-(OG)$_6$** S12

Table S3: Extracted data from the MALDI mass spectrum of **TBTQ-(OG)$_6$** S13

Figure S15: ESI-HRMS spectrum of **TBTQ-(OG)$_6$**... S14

Table S4: Abundance pattern of the [M – 2 H]$^{2-}$ ions from the
ESI mass spectrum of **TBTQ-(OG)$_6$** (low resolution) S14

Figure S16: Molar ratio plot for C_{60}/C_{70} and **TBTQ(OG)$_6$** in toluene-DMSO................. S15

Figure S17: Optical images of C_{60}, **TBTQ-(OG)$_6$** ⊂ C_{60}, C_{70} and **TBTQ-(OG)$_6$** ⊂ C_{70}
dispersed in water at different times... S15

Table S5: *Xyz* coordinates of **TBTQ-(OG)$_6$** ⊂ C_{60} complex... S16

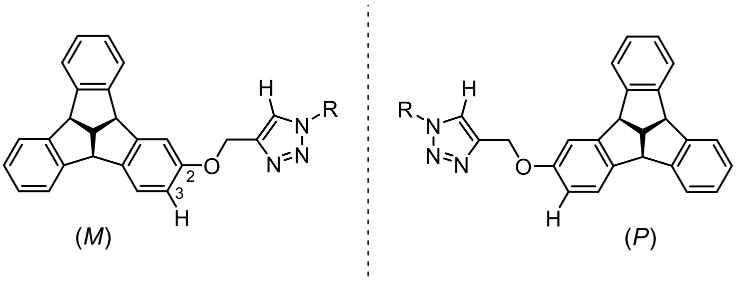

Figure S1: Enantiotopic triazole protons in parent (*M*)- and (*P*)-enantiomers (R = H).

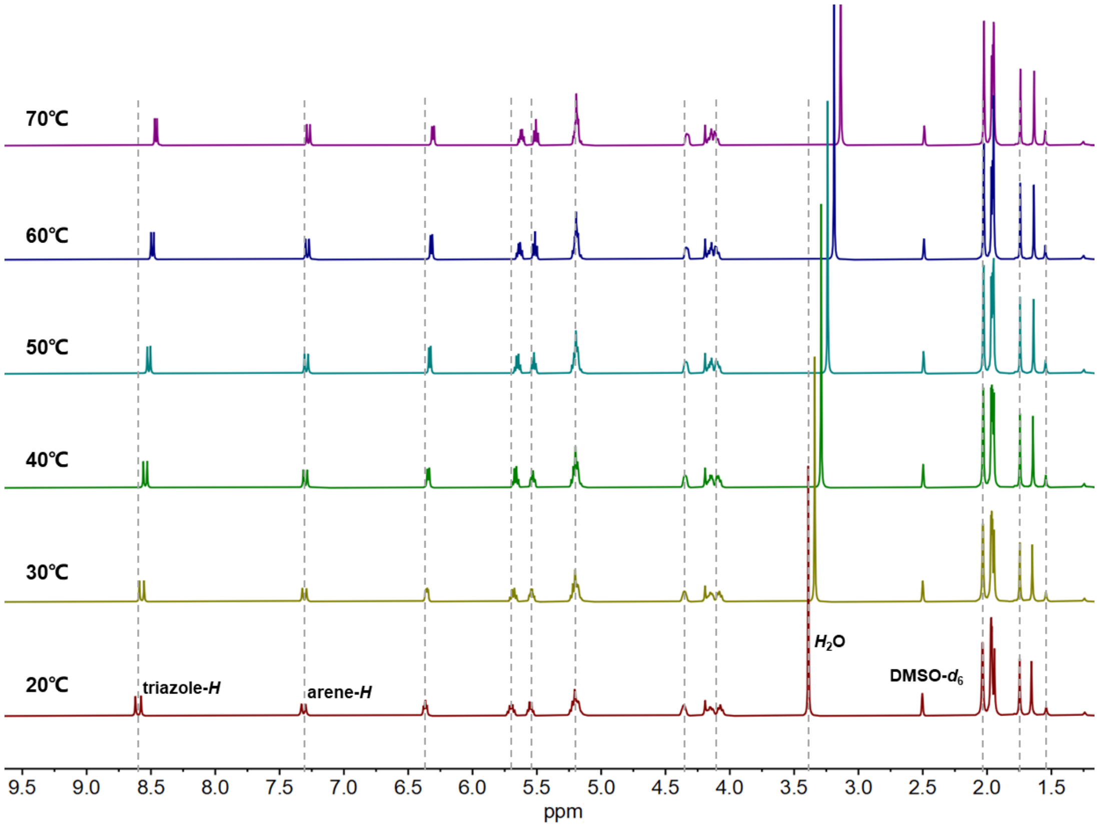

Figure S2: ^{1}H NMR spectra of **TBTQ-(OAcG)$_6$** at different temperatures (DMSO-d_6, 400 MHz).

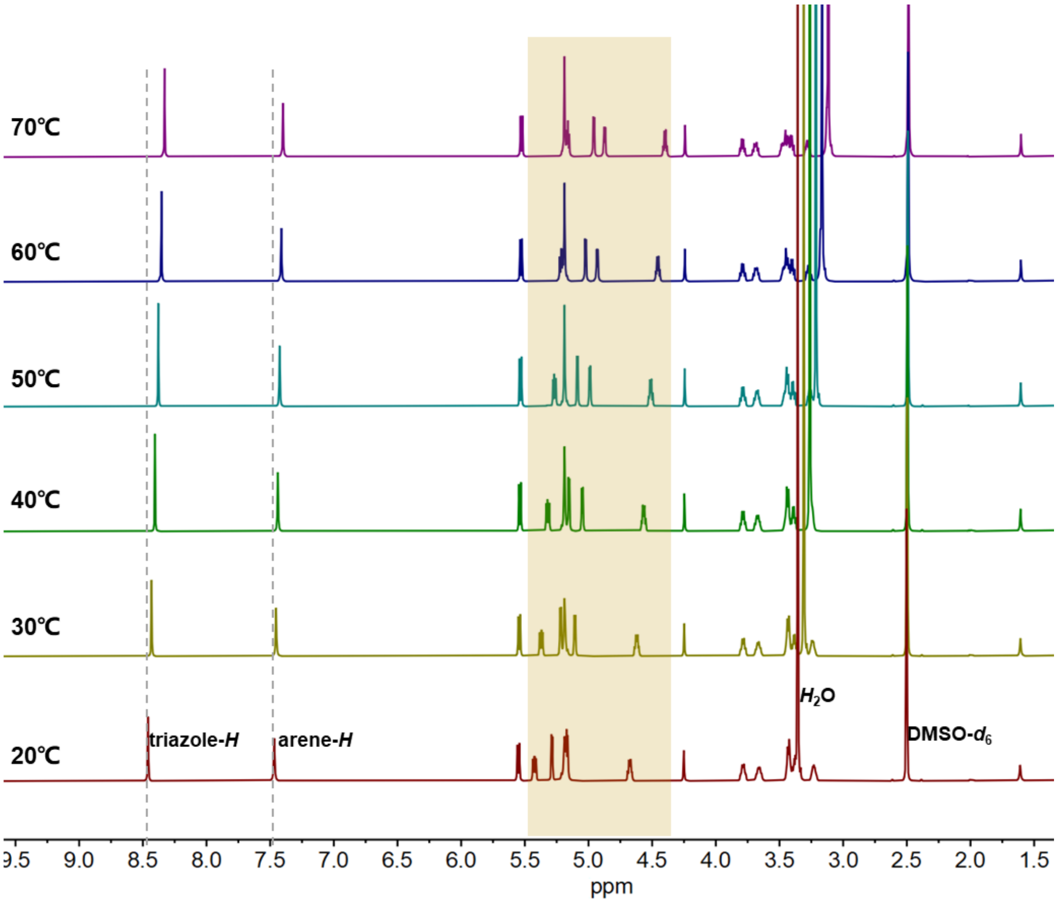

Figure S3: ^{1}H NMR spectra of **TBTQ-(OG)$_6$** at different temperatures (DMSO-d_6, 400 MHz).

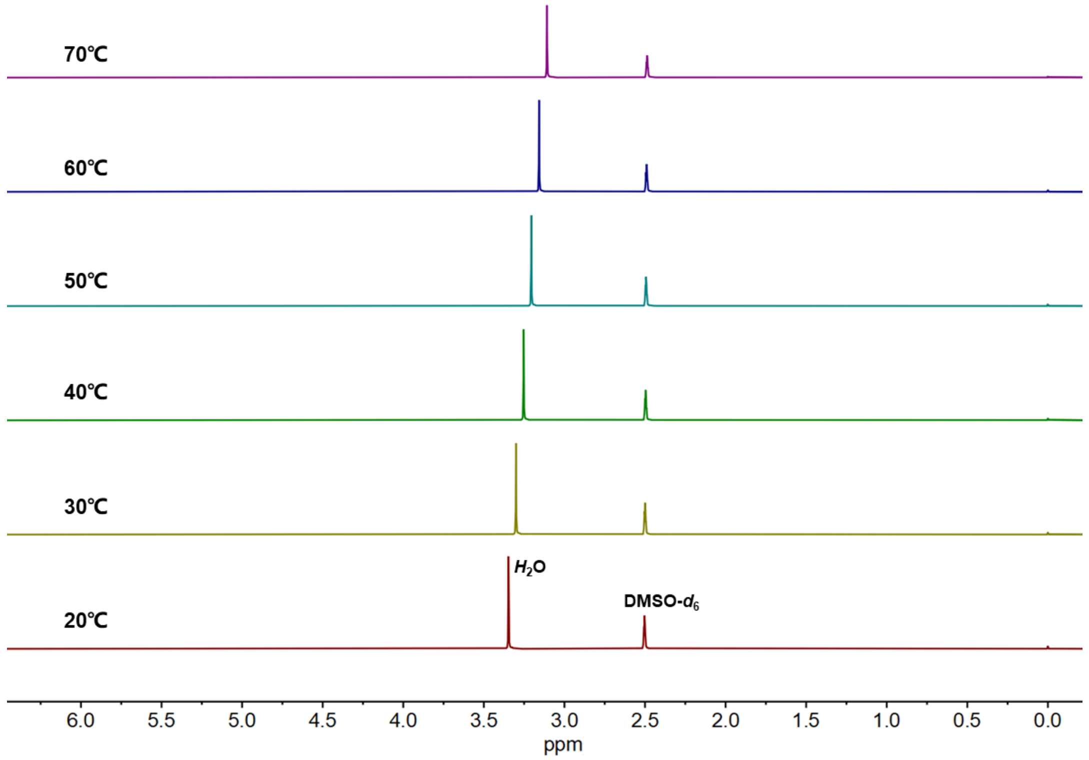

Figure S4: Blank ^{1}H NMR spectra of water in DMSO-d_6 (400 MHz) at different temperatures.

Note: The signal of the water protons shows the same chemical shift changes as does the corresponding signal in the spectra of Figures S2 and S3. This proves that these shifts are independent of the presence of **TBTQ-(OAcG)$_6$** and **TBTQ-(OG)$_6$**.

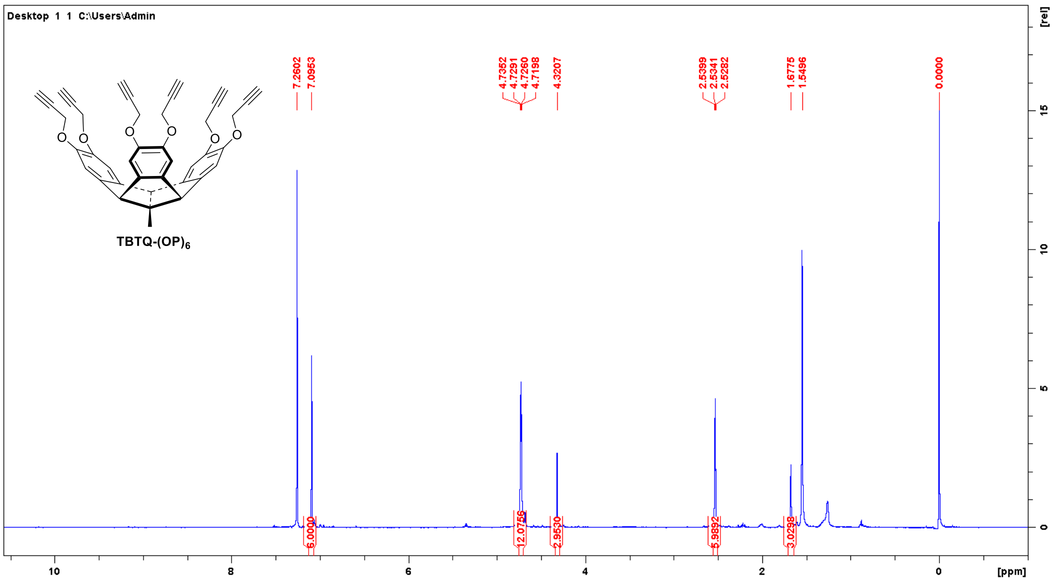

Figure S5: ^{1}H NMR spectrum of **TBTQ-(OP)$_6$** (400 MHz, $CDCl_3$).

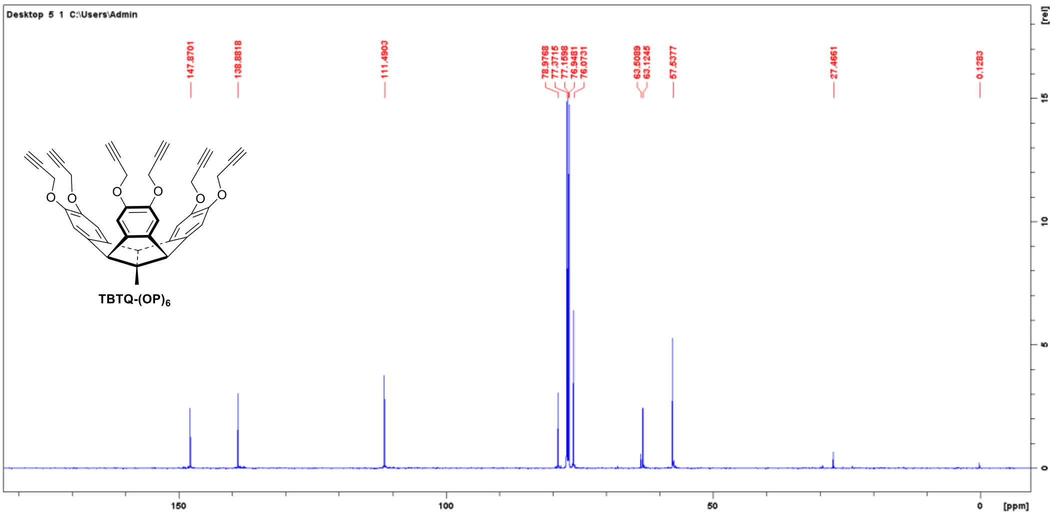

Figure S6: ^{13}C NMR spectrum of **TBTQ-(OP)$_6$** (100 Mz, $CDCl_3$).

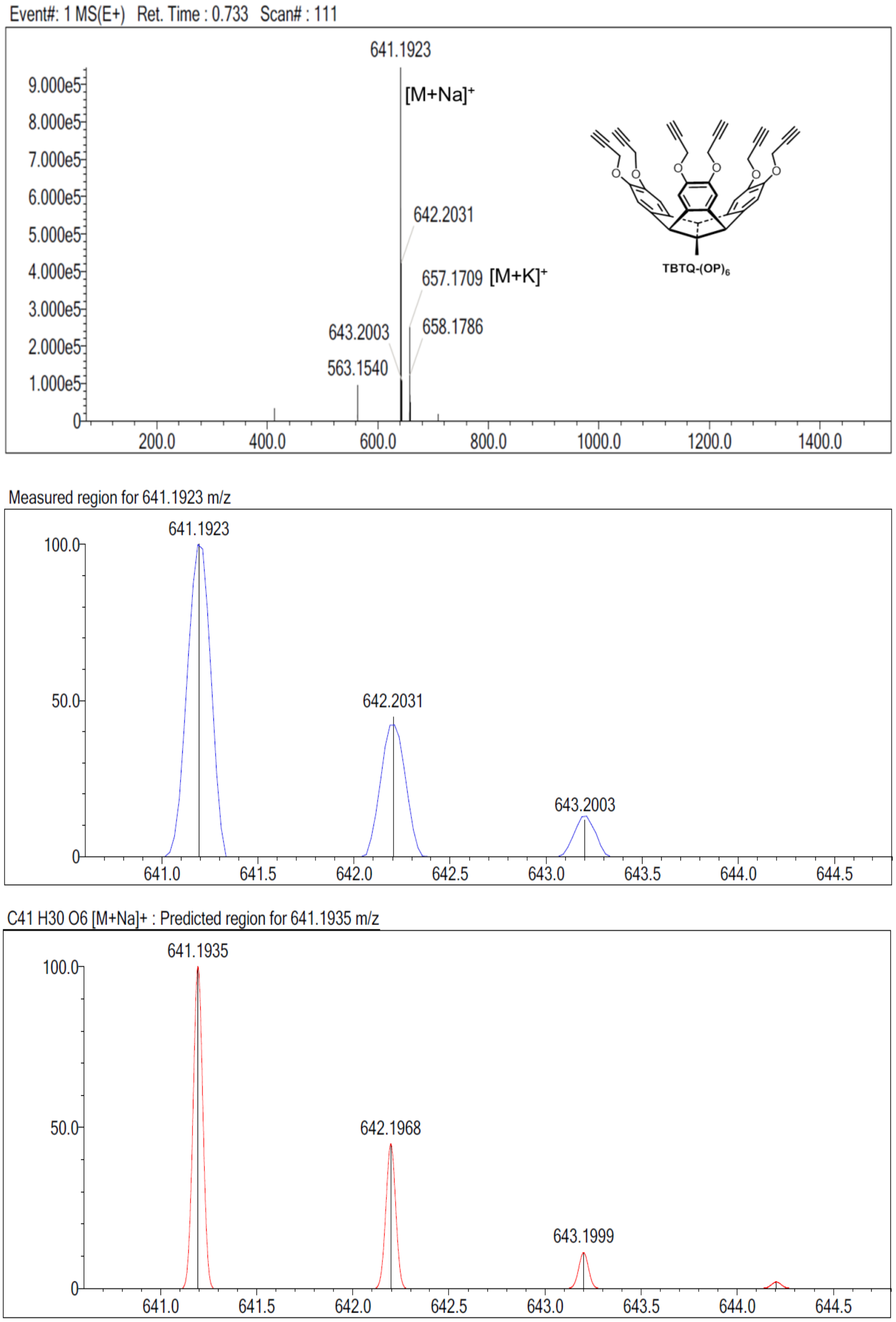

Rank	Score	Formula (M)	Ion	Meas. m/z	Pred. m/z	Df. (mDa)	Df. (ppm)	Iso	DBE
2	90.81	C41 H30 O6	[M+Na]+	641.1923	641.1935	-1.2	-1.87	92.83	27.0

Figure S7: ESI-HRMS spectrum of **TBTQ-(OP)$_6$**.

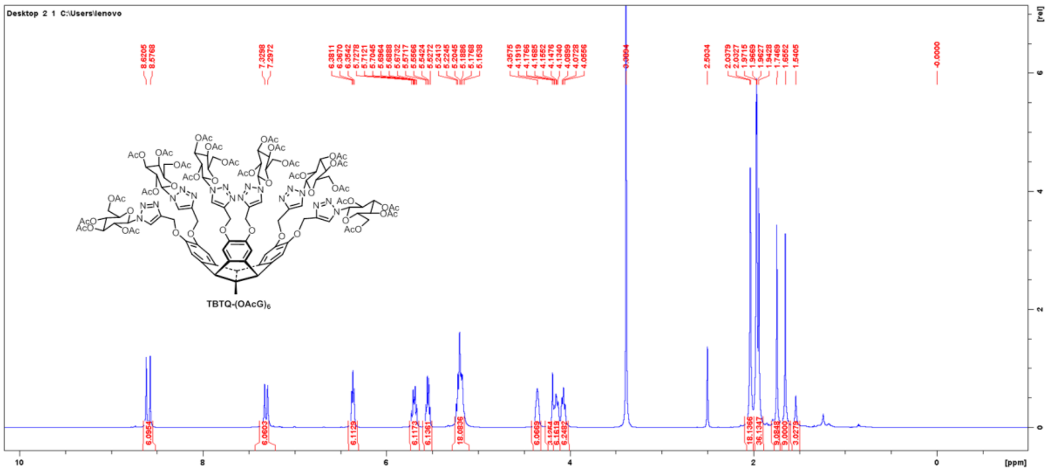

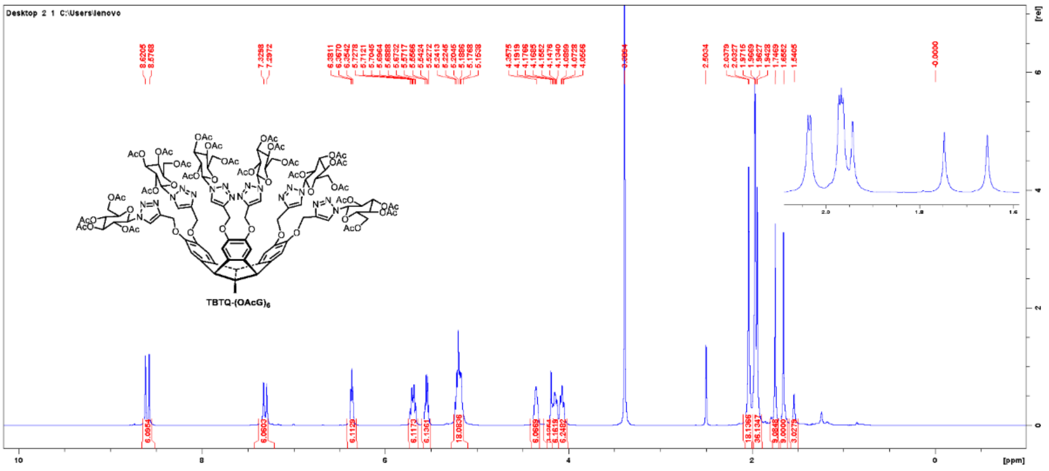

Figure S8: ^{1}H NMR spectrum of **TBTQ-(OAcG)$_6$** (400 MHz, DMSO-d_6).

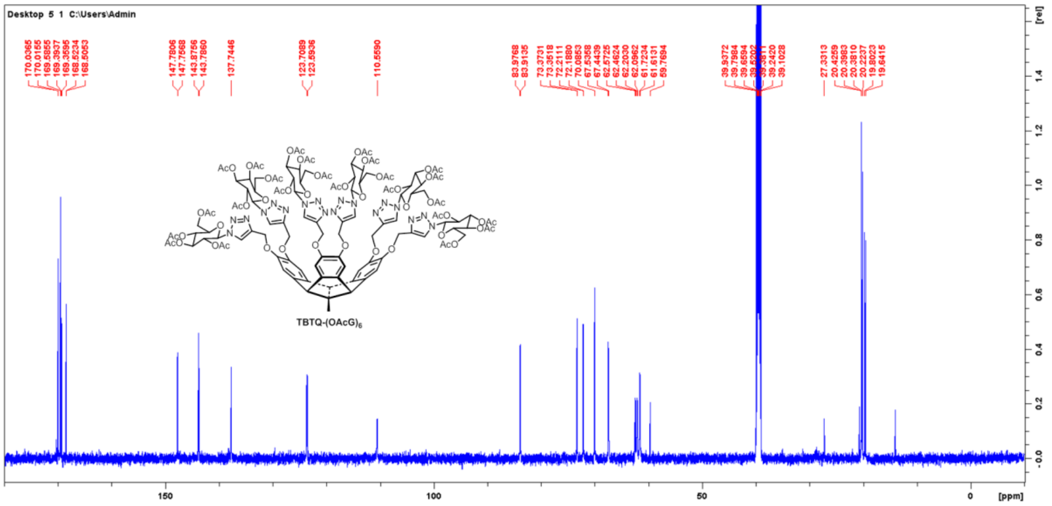

Figure S9: ^{13}C NMR spectrum of **TBTQ-(OAcG)$_6$** (100 MHz, DMSO-d_6).

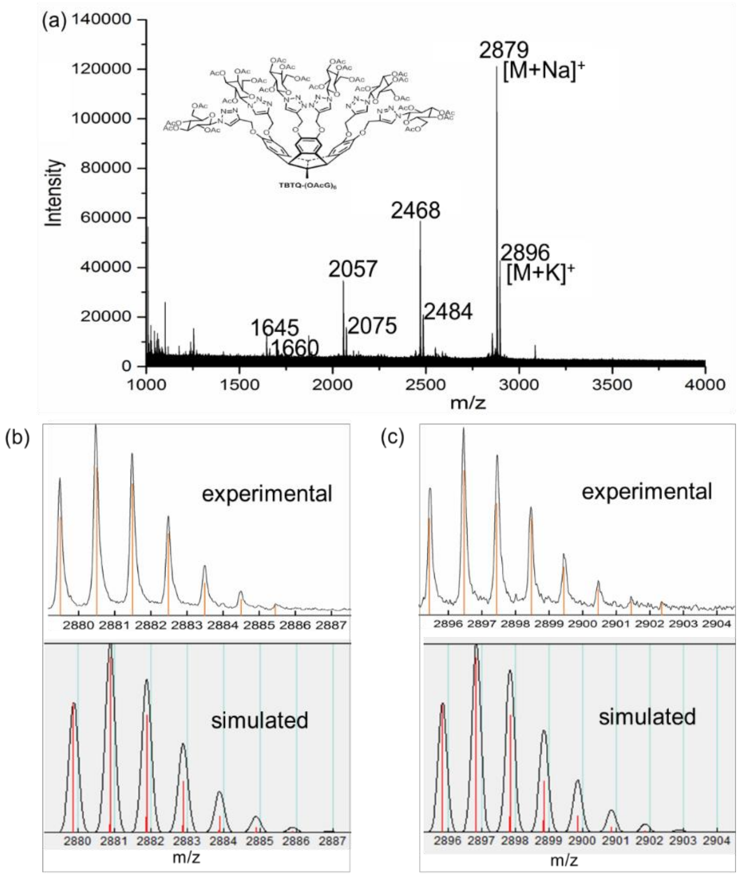

Figure S10: MALDI mass spectrum of **TBTQ-(OAcG)$_6$**: (a) full spectrum, (b) isotopic distribution of the [M + Na]$^+$ molecular adduct ions (top: experimental, bottom: simulated), (c) isotopic distribution of the [M + K]$^+$ molecular adduct ions (top: experimental, bottom: simulated).

Table S1: Extracted data from MALDI mass spectrum of **TBTQ-(OAcG)$_6$**.

Na$^+$ adduct ions	*m*/*z*	Intensity	K$^+$ adduct ions	*m*/*z*	Intensity
[M + Na]$^+$	**2879.479**	87414	[M + K]$^+$	**2895.450**	29538
	2880.471	121107		2896.457	42732
	2881.476	102767		2897.452	36721
[M + Na – Tcle]$^{+\bullet}$	**2468.404**	47195	[M + K – Tcle]$^{+\bullet}$	**2484.372**	19272
	2469.406	58681		2485.377	20901
	2470.408	40665		2486.371	16783
[M + Na – 2Tcle]$^+$	**2057.336**	34724	[M + K – 2 Tcle]$^+$	**2073.310**	15908
	2058.327	34118		2074.316	15173
	2059.339	21604		2075.310	12577
[M + Na – 3 Tcle]$^{+\bullet}$	**1645.248**	9364	[M + K – 3 Tcle]$^{+\bullet}$	**1661.250**	5869
	1646.269	13563		1662.257	7323
	1647.262	10072		1663.245	6040
	1648.265	7480		1664.233	769

Comment: In most cases, the mass difference is 411 u. This does not simply correspond to the loss of the tentacle-like residue, “Tcle”, but to subsequent addition of a H atom to the remaining phenoxy-type fragment ion, leading to a fragment ions [M + Na – n · Tcle + H]$^+$ (n = 1–3). It is well possible that this reduction process happens in the matrix under laser irradiation. – The *m*/*z* values of the monoisotopic ions are given in boldface.

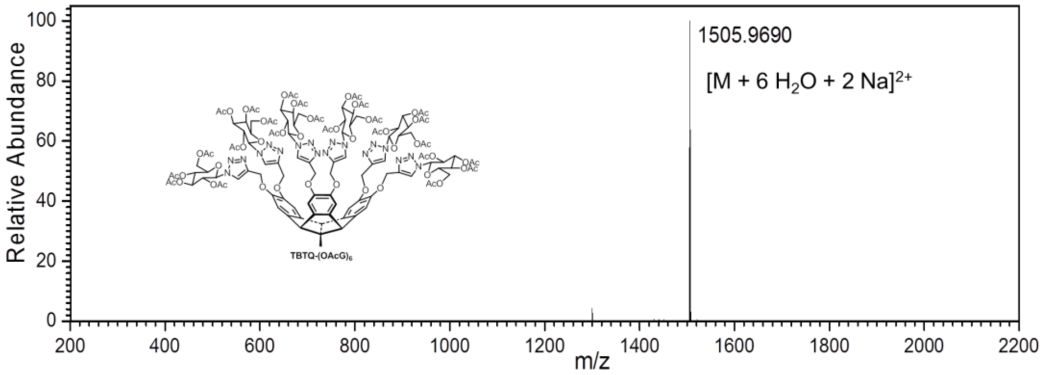

Figure S11: ESI-HRMS spectrum of **TBTQ-(OAcG)$_6$.** The peak labeled “1505.9690” corresponds to the [M + 1] ions (mainly the $^{13}C_1$-isotopolog).

Table S2: Abundance pattern of the $[M + 6\ H_2O + 2\ Na]^{2+}$ ions from the ESI mass spectrum of **TBTQ-(OAcG)$_6$** (low resolution).

m/*z*	1505.5	1506.0	1506.5	1507.0	1507.5	1508.0	1508.5	1509.0
Calc’d (% B)	68.5	100.0	81.8	48.4	22.9	9.2	3.2	1.0
Exp’l (% B)	58	100	94	64	23	4	1	< 1

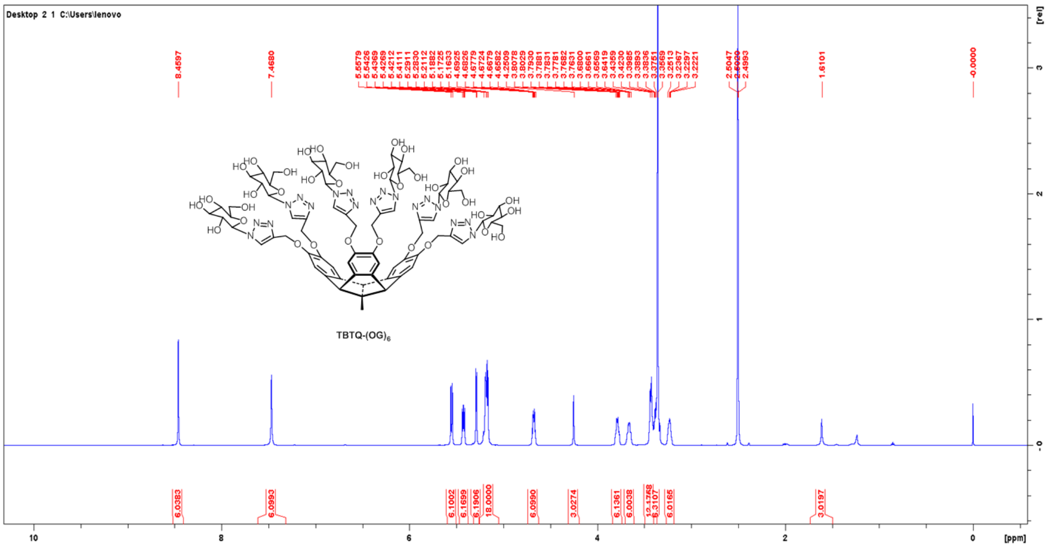

Figure S12: ^{1}H NMR spectrum of **TBTQ-(OG)$_6$** (400 MHz, DMSO-d_6).

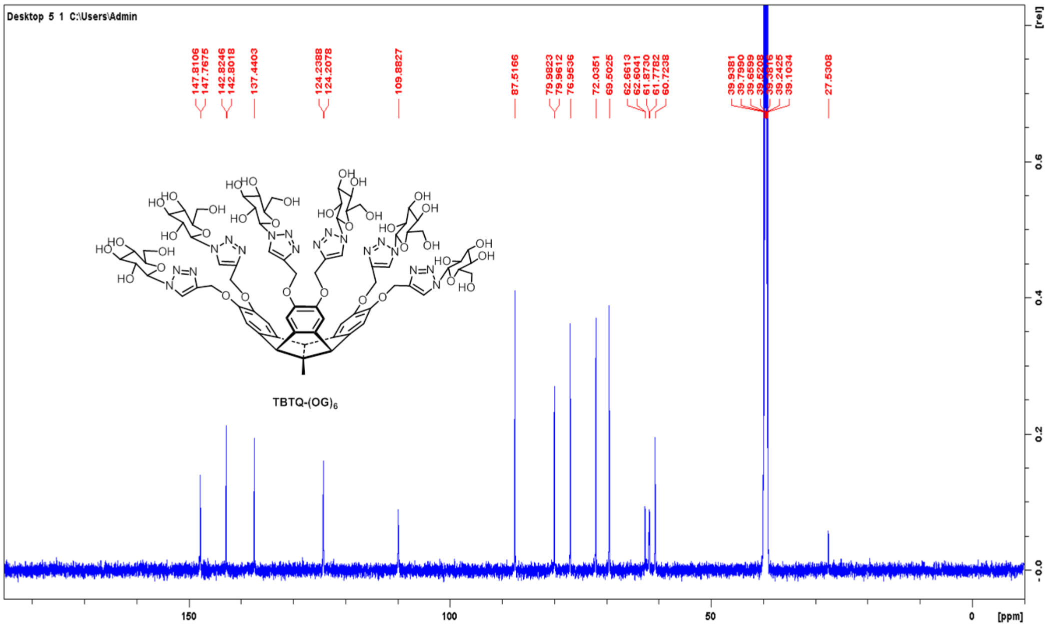

Figure S13: ^{13}C NMR spectrum of **TBTQ-(OG)$_6$** (100 MHz, DMSO-d_6).

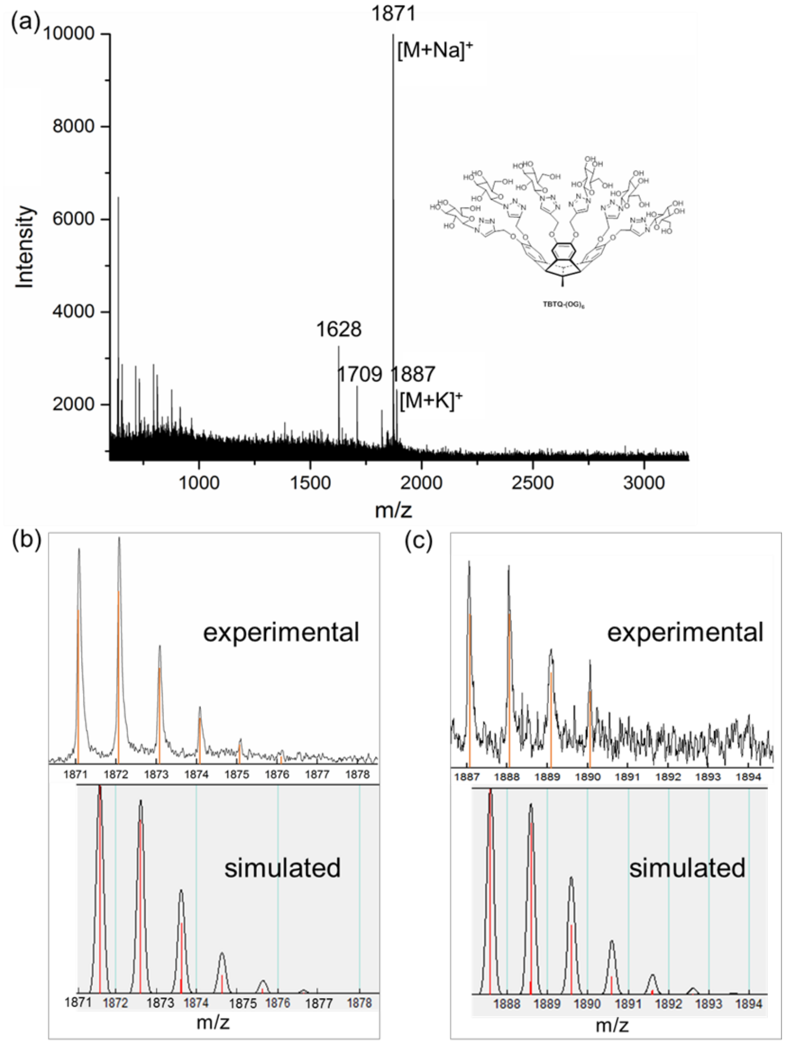

Figure S14: MALDI mass spectrum of **TBTQ-(OG)$_6$**. (a) full spectrum, (b) isotopic distribution of [M + Na]$^+$ molecular adduct ions (top: experimental, bottom: simulated), (c) isotopic distribution of [M + K]$^+$ molecular adduct ions (top: experimental, bottom: simulated).

Table S3: Extracted data from MALDI mass spectrum of **TBTQ-(OG)$_6$**.

Na^+ adduct ions	*m*/*z*	Intensity	K^+ adduct ions	*m*/*z*	Intensity
$[M + Na]^+$	**1871.085**	11032	$[M + K]^+$	**1887.057**	2330
	1872.082	11573		1888.038	2295
	1873.090	6428		---	---
$[M + Na – C_6H_{10}O_5]^+$	**1709.086**	2311	---	---	---
	1710.098	2404			
	---	---			
$[M + Na – Tcle]^{+\bullet}$ =	**1628.080**	3268	---	---	---
$[M + Na – C_9H_{14}N_3O_5]^{+\bullet}$	1629.078	2775			
	1630.056	1726			

Comment: Here, the observed mass differences are 162 u and 243 u. In the first case, this corresponds to the loss of glycosyl residue (as a radical); in the second case, this corresponds to the loss of the entire tentacle (as a radical) with subsequent transfer of a H atom, probably from the matrix to the remaining phenoxy-type fragment ion, leading to a fragment ions $[M + Na – Tcle + H]^+$. It is assumed that this reduction process happens in the matrix under laser irradiation. – The *m*/*z* values of the monoisotopic ions are given in boldface.

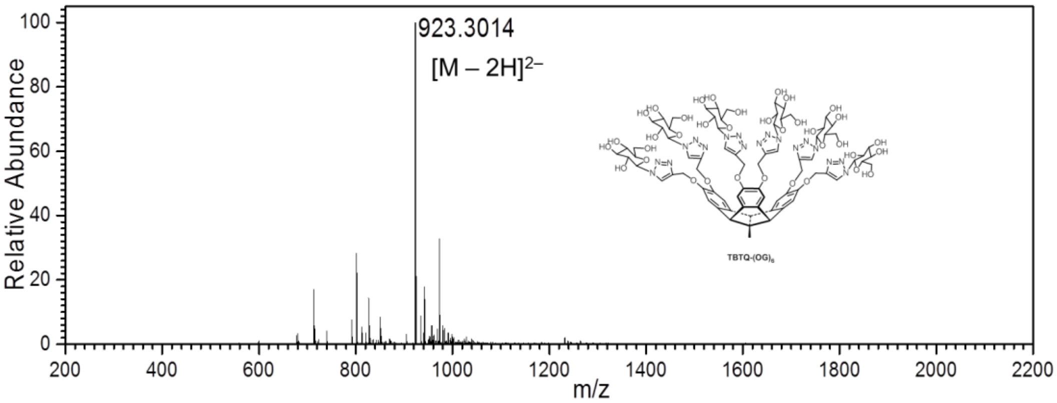

Figure S15: ESI-HRMS (negative mode) mass spectrum of **TBTQ-(OG)$_6$**. The peak labeled "923.3014" corresponds to the monoisotopic ion.

Table S4: Abundance pattern of the $[M – 2\ H]^{2–}$ ions from the ESI mass spectrum of **TBTQ-(OG)$_6$** (low resolution).

m/z	923.3	923.8	924.3	924.8	925.3	925.8	926.3
Calc'd (% B)	100.0	**92.3**	49.5	19.5	6.2	1.7	0.4
Exp'l (% B)	100	98	55	21	8	1	< 1

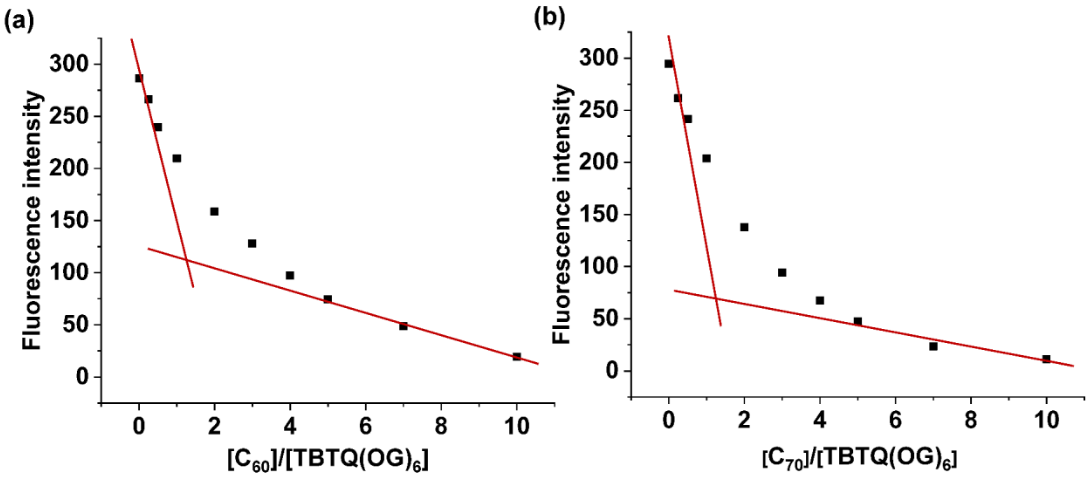

Figure S16: (a) Molar ratio plot for C_{60} and **TBTQ(OG)$_6$** in toluene/DMSO 1:1 (v/v), indicating 1:1 stoichiometry; (b) molar ratio plot for C_{70} and **TBTQ(OG)$_6$** in toluene/DMSO 1:1 (v/v), indicating 1:1 stoichiometry.

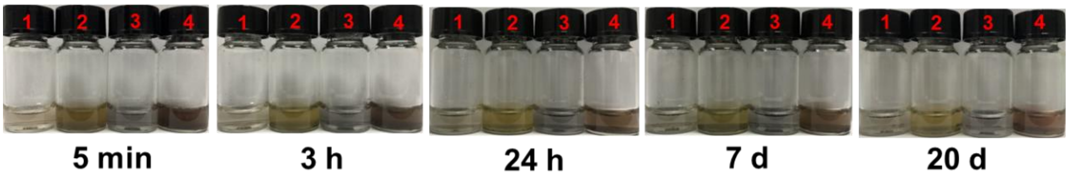

Figure S17: Optical images of **1**: C_{60}, **2**: **TBTQ-(OG)$_6$** $\subset$ C_{60}, **3**: C_{70} and **4**: **TBTQ-(OG)$_6$** $\subset$ C_{70} dispersed in water at different times without centrifugation [C_{60}: 2 mM; C_{70}: 2 mM; **TBTQ-(OG)$_6$**: 20 mM].

Table S5: The *xyz* coordinates (in Å) of **TBTQ-(OG)$_6$** ⊂ C_{60} complex at the B3LYP/6-31G(d) level of theory.

The number of imaginary frequencies: 0

Total Energy (optimized structures)：–8970.94451731 a.u.

Optimized Coordinates: (1-227：**TBTQ-(OG)$_6$**；228-287：C_{60})

1	C	0.531	-2.337	5.919
2	C	0.120	-0.943	6.542
3	C	1.860	-2.091	5.259
4	C	-0.627	-2.719	5.041
5	C	1.238	0.084	6.120
6	C	2.277	-0.752	5.423
7	C	-1.205	-0.500	5.806
8	C	-1.624	-1.714	5.020
9	C	0.539	1.067	5.220
10	C	1.077	2.227	4.678
11	C	0.242	3.059	3.917
12	C	-1.103	2.685	3.665
13	C	-1.636	1.505	4.219
14	C	-0.815	0.717	5.011
15	C	-0.845	-3.947	4.431

16	C	-2.119	-4.215	3.909
17	C	-3.144	-3.239	3.967
18	C	-2.875	-1.952	4.460
19	C	3.548	-0.344	5.038
20	C	4.422	-1.304	4.510
21	C	3.984	-2.631	4.267
22	C	2.688	-3.027	4.653
23	C	-0.057	-1.018	8.046
24	O	0.614	4.308	3.448
25	O	-2.037	3.407	2.954
26	O	5.754	-1.069	4.235
27	O	4.679	-3.649	3.645
28	O	-4.384	-3.707	3.588
29	O	-2.383	-5.522	3.513
30	C	6.265	1.203	3.672
31	C	6.932	1.256	2.441
32	N	6.561	2.491	1.900
33	N	5.687	3.129	2.760
34	N	5.500	2.381	3.800
35	C	6.907	2.789	-2.241
36	C	6.196	4.004	-1.611
37	C	5.951	3.856	-0.099

38	C	7.084	3.126	0.666
39	O	7.621	1.997	-0.050
40	C	8.093	2.318	-1.382
41	C	8.681	0.983	-1.859
42	O	7.744	-0.091	-1.706
43	O	5.966	5.194	0.406
44	O	4.936	4.056	-2.296
45	O	7.525	3.192	-3.472
46	C	6.359	0.159	4.716
47	C	6.524	-3.322	2.129
48	C	6.495	-4.209	1.041
49	N	7.082	-3.502	-0.014
50	N	7.426	-2.242	0.421
51	N	7.108	-2.123	1.673
52	C	6.195	-4.494	-4.006
53	C	7.368	-3.502	-3.868
54	C	7.508	-2.937	-2.441
55	C	7.372	-4.044	-1.365
56	O	6.215	-4.871	-1.603
57	C	6.228	-5.552	-2.886
58	C	4.955	-6.409	-2.851
59	O	5.183	-7.582	-2.071

60	O	8.847	-2.452	-2.355
61	O	7.003	-2.433	-4.754
62	O	6.347	-5.256	-5.207
63	C	6.128	-3.583	3.536
64	C	2.109	5.105	1.817
65	C	1.373	6.111	1.168
66	N	1.865	6.125	-0.141
67	N	2.863	5.189	-0.257
68	N	3.019	4.583	0.882
69	C	-1.034	7.890	-2.548
70	C	-0.438	6.589	-3.160
71	C	0.638	5.908	-2.290
72	C	1.430	6.881	-1.364
73	O	0.766	8.045	-0.939
74	C	-0.671	8.090	-1.059
75	C	-1.398	7.099	-0.149
76	O	-1.586	7.639	1.156
77	O	1.596	5.412	-3.225
78	O	-1.528	5.709	-3.421
79	O	-2.461	7.889	-2.627
80	C	2.030	4.573	3.201
81	C	-1.531	4.390	2.000

82	C	-5.546	-2.847	3.739
83	C	-5.863	-2.290	2.402
84	N	-7.174	-2.189	1.895
85	N	-7.129	-1.761	0.670
86	N	-5.817	-1.562	0.310
87	C	-4.986	-1.877	1.393
88	C	-5.966	-0.421	-3.782
89	C	-6.651	-1.701	-3.242
90	C	-5.841	-2.334	-2.088
91	C	-5.386	-1.249	-1.065
92	O	-6.033	0.011	-1.334
93	C	-5.689	0.583	-2.637
94	C	-6.636	1.790	-2.674
95	O	-6.451	2.592	-1.492
96	O	-4.650	-2.861	-2.665
97	O	-7.954	-1.379	-2.775
98	O	-4.742	-0.718	-4.437
99	C	-3.610	-5.452	1.433
100	N	-3.741	-4.672	0.268
101	N	-4.993	-4.619	-0.074
102	N	-5.750	-5.315	0.838
103	C	-4.896	-5.863	1.807

104	C	-8.513	-6.904	-1.584
105	C	-8.412	-5.364	-1.624
106	C	-7.938	-4.719	-0.309
107	C	-7.207	-5.637	0.703
108	O	-7.280	-7.032	0.479
109	C	-7.308	-7.545	-0.870
110	C	-5.990	-7.339	-1.630
111	O	-4.930	-8.021	-0.965
112	O	-9.131	-4.305	0.358
113	O	-7.429	-4.950	-2.587
114	O	-8.431	-7.364	-2.944
115	C	-2.314	-5.768	2.085
116	C	-2.551	4.455	0.929
117	N	-2.224	4.216	-0.427
118	N	-3.267	4.444	-1.166
119	N	-4.315	4.828	-0.370
120	C	-3.895	4.838	0.970
121	C	-7.171	7.523	-0.060
122	C	-7.225	6.250	0.809
123	C	-6.787	5.012	0.009
124	C	-5.612	5.252	-0.976
125	O	-5.449	6.560	-1.491

126	C	-5.785	7.724	-0.706
127	C	-4.708	8.090	0.320
128	O	-3.675	8.886	-0.262
129	O	-7.941	4.677	-0.762
130	O	-6.299	6.325	1.905
131	O	-7.438	8.579	0.873
132	H	0.659	-3.111	6.714
133	H	1.678	0.604	7.000
134	H	-2.003	-0.224	6.535
135	H	2.121	2.489	4.848
136	H	-2.672	1.243	4.017
137	H	-0.076	-4.718	4.397
138	H	-3.633	-1.173	4.445
139	H	3.855	0.695	5.145
140	H	2.370	-4.053	4.481
141	H	-0.837	-1.738	8.325
142	H	0.869	-1.329	8.546
143	H	-0.343	-0.047	8.468
144	H	7.569	0.536	1.946
145	H	6.187	1.962	-2.445
146	H	6.743	4.955	-1.825
147	H	4.959	3.373	0.091

148	H	7.918	3.830	0.923
149	H	8.877	3.107	-1.322
150	H	9.602	0.733	-1.302
151	H	8.871	1.015	-2.954
152	H	7.407	-0.124	-0.767
153	H	5.342	5.252	1.177
154	H	4.302	4.629	-1.792
155	H	6.828	3.527	-4.086
156	H	5.926	0.472	5.684
157	H	7.409	-0.176	4.858
158	H	6.104	-5.206	0.947
159	H	5.229	-3.937	-4.046
160	H	8.330	-3.943	-4.217
161	H	6.790	-2.097	-2.277
162	H	8.292	-4.681	-1.322
163	H	7.137	-6.191	-2.956
164	H	4.729	-6.803	-3.867
165	H	4.090	-5.849	-2.450
166	H	5.340	-7.326	-1.135
167	H	8.818	-1.577	-1.851
168	H	7.642	-1.688	-4.657
169	H	6.393	-4.638	-5.977

170	H	6.566	-2.846	4.239
171	H	6.415	-4.612	3.845
172	H	0.604	6.782	1.538
173	H	-0.710	8.779	-3.140
174	H	-0.026	6.780	-4.187
175	H	0.184	5.061	-1.715
176	H	2.355	7.266	-1.882
177	H	-0.864	9.143	-0.729
178	H	-0.816	6.178	0.023
179	H	-2.390	6.845	-0.582
180	H	-2.240	8.394	1.095
181	H	2.140	4.692	-2.815
182	H	-1.720	5.101	-2.664
183	H	-2.789	6.992	-2.973
184	H	2.661	3.667	3.328
185	H	2.297	5.334	3.960
186	H	-1.437	5.351	2.552
187	H	-0.536	4.087	1.609
188	H	-6.319	-3.566	4.086
189	H	-5.395	-2.065	4.506
190	H	-3.907	-1.832	1.389
191	H	-6.585	0.038	-4.589

192	H	-6.817	-2.447	-4.056
193	H	-6.446	-3.159	-1.616
194	H	-4.277	-1.117	-1.097
195	H	-4.615	0.879	-2.609
196	H	-7.698	1.475	-2.710
197	H	-6.402	2.476	-3.511
198	H	-6.324	1.992	-0.709
199	H	-4.324	-3.633	-2.131
200	H	-7.892	-0.872	-1.919
201	H	-4.252	-1.449	-3.967
202	H	-5.225	-6.438	2.656
203	H	-9.465	-7.225	-1.108
204	H	-9.385	-4.903	-1.927
205	H	-7.306	-3.827	-0.588
206	H	-7.680	-5.541	1.724
207	H	-7.472	-8.632	-0.660
208	H	-5.739	-6.269	-1.764
209	H	-6.041	-7.830	-2.625
210	H	-4.787	-7.613	-0.083
211	H	-8.996	-3.417	0.778
212	H	-7.405	-5.606	-3.336
213	H	-9.324	-7.402	-3.350

214	H	-2.111	-6.862	2.067
215	H	-1.463	-5.224	1.637
216	H	-4.532	5.104	1.805
217	H	-7.967	7.482	-0.841
218	H	-8.253	6.106	1.223
219	H	-6.551	4.172	0.708
220	H	-5.761	4.655	-1.930
221	H	-5.814	8.493	-1.520
222	H	-4.258	7.199	0.795
223	H	-5.123	8.749	1.113
224	H	-3.375	8.501	-1.143
225	H	-7.797	3.779	-1.194
226	H	-6.461	7.167	2.402
227	H	-7.577	9.430	0.406
228	C	-0.204	-4.169	-1.105
229	C	-1.277	-1.913	-3.689
230	C	-1.087	-3.561	-1.982
231	C	-1.984	-2.506	-1.498
232	C	-1.021	-2.769	0.764
233	C	-0.170	-3.761	0.306
234	C	-2.038	-0.700	0.191
235	C	-1.952	-2.123	-0.168

236	C	-0.647	-3.193	-3.335
237	C	-2.102	-1.488	-2.553
238	C	-0.554	1.719	0.377
239	C	-1.163	-0.471	1.347
240	C	-0.437	0.706	1.432
241	C	1.707	1.680	1.109
242	C	2.911	-3.499	-3.006
243	C	0.298	2.441	-2.252
244	C	-1.386	1.502	-0.708
245	C	1.398	-2.456	-4.510
246	C	3.441	1.777	-0.646
247	C	3.383	0.996	-3.392
248	C	2.529	1.987	-2.936
249	C	-0.574	-0.969	-4.421
250	C	4.399	-1.072	-2.819
251	C	2.058	-4.217	-0.377
252	C	3.007	1.415	0.710
253	C	3.307	-3.650	-0.567
254	C	1.182	2.670	-1.100
255	C	2.796	-2.484	-4.061
256	C	2.559	2.386	-1.524
257	C	4.349	0.728	-1.129

258	C	1.570	0.001	-4.748
259	C	1.175	-4.449	-1.528
260	C	2.935	-0.804	1.794
261	C	1.588	-4.101	-2.804
262	C	4.506	-2.028	-1.823
263	C	4.317	0.348	-2.462
264	C	3.016	-2.226	1.432
265	C	-0.950	1.875	-2.061
266	C	-0.660	0.453	-4.064
267	C	4.472	-0.285	-0.071
268	C	0.771	2.321	0.177
269	C	-0.532	-1.749	1.702
270	C	1.226	-3.793	0.758
271	C	3.643	0.138	1.065
272	C	0.653	-3.456	-3.734
273	C	0.961	0.679	1.884
274	C	4.544	-1.625	-0.413
275	C	1.131	2.017	-3.386
276	C	-2.152	0.257	-0.804
277	C	1.555	-0.526	2.219
278	C	2.889	-0.027	-4.326
279	C	0.666	1.053	-4.266

280	C	3.520	-1.305	-3.972
281	C	-1.445	0.854	-2.995
282	C	1.691	-2.828	1.637
283	C	3.741	-3.276	-1.919
284	C	0.788	-1.776	2.123
285	C	0.804	-1.249	-4.844
286	C	3.802	-2.627	0.364
287	C	-2.187	-0.147	-2.217
